# Enhanced mechanical, thermal, and wear performance of halloysite nanotube infused carbon fiber epoxy composites

**DOI:** 10.1038/s41598-025-15142-1

**Published:** 2025-08-16

**Authors:** Muralidhara B, S. P. Kumaresh Babu, Suresha B, Yogesha K K, Vasundhara M G, Kalavathi G K, Dayanand M. Goudar, Rajashekar V. Kurahatti, Subraya Krishna Bhat

**Affiliations:** 1 Department of Metallurgical & Materials Engineering, National Institute of Technology, Tiruchirappalli, India; 2 Department of Mechanical Engineering, Government Engineering College, Mosalehosahalli, 573212 India; 3 Department of Mechanical Engineering, JSS Science and Technology University, Mysuru, India; 4 Department of Mechanical Engineering, The National Institute of Engineering, Mysuru, India; 5 Department of Mechanical Engineering, Malnad College of Engineering, Hassan, 573202 India; 6 Department of Mathematics, Malnad College of Engineering, Hassan, Karnataka 573202 India; 7 Department of Mechanical Engineering, Tontadarya College of Engineering, Gadag, 582 101 India; 8 Department of Mechanical Engineering, Basaveshwar Engineering College, Bagalkote, 587 101 India; 9 Department of Mechanical and Industrial Engineering, Manipal Institute of Technology, Manipal Academy of Higher Education, Manipal, Karnataka 576104 India

**Keywords:** Carbon/Epoxy composite, Halloysite nanotubes, Mechanical behaviour, Thermal properties, Wear, Scanning electron microscopy, Mechanical engineering, Engineering, Materials science

## Abstract

This work explores the mechanical, thermal, and tribological characteristics of carbon fabric reinforced epoxy (CF-Ep) composites filled with halloysite nanotubes (HNT). The mechanical properties were evaluated, including hardness, interlaminar shear strength (ILSS), tensile strength, and flexural strength. The enhanced curing and even dispersion of HNTs in the epoxy matrix were validated by DSC, FTIR, and SEM measurements. Thermogravimetric analysis (TGA) and dynamic mechanical analysis (DMA) demonstrated improved thermal stability and damping, especially for the 0.75 wt.% HNT composite. Tribological performance was investigated utilizing a pin-on-disc configuration (60 N, 3 m/s) and silica sand (212 µm, 30 N, 2.38 m/s) under three-body abrasion and dry sliding wear, respectively. Hardness was highest at 1.75 wt.% HNT, and wear resistance and mechanical performance were best at 0.75 wt.% HNT composite. Surface damage, including matrix separation, micro-ploughing, and fragmentation, was lessened in 0.75 wt.% HNT composites, according to scanning electron microscopy worn surfaces. Using Minitab 17 and the Taguchi approach, wear experiments was created using an L16 orthogonal array. There were four levels of variation in three factors: load (10–40 N), abrading distance (250–1000 m), and HNT content (0–2.75 wt.%). The most important variables influencing wear volume loss were found to be load + distance and load + filler interactions using ANOVA and regression analysis. Scanning electron microscopy revealed that H0.75% HNT-filled composites had the best resistance to wear because they showed less surface damage mechanisms, such as fragmentation, micro-ploughing, and matrix detachment, when they were worn-out by dry sliding or abrasion. Overall, by strengthening interfacial bonding, improving load transfer, and creating a protective tribolayer that decreased material loss and surface damage during abrasion, HNTs improved mechanical and wear properties. Specifically, the 0.75 wt.% HNT composite showed outstanding heat stability and wear resistance, which made it a good option for high-performance uses such as power plant chute and automobile liners.

## Introduction

The nanostructure control and nanoparticle addition to polymers and the structural and functional behavior enhancement have led the polymeric systems as a continuous requirement for advanced industrial sectors. The full potential of these materials is contingent upon the successful up scaling and enhancement of efficient manufacturing processes. Their distinct bulk and surface characteristics have drawn a lot of attention to these nanomaterials, demonstrating that not all polymers are equally suited for use at the nanoscale. For effective nano structuration and nano compounding, the polymer matrix and nanoparticles must be carefully designed^1^. Nanosized particles leverage their high surface-to-volume ratio (S/V) for enhanced filler–polymer interaction, but their effectiveness is often compromised by agglomeration issues^2^. The composition of the matrix and its microstructure play a crucial role in determining the fiber/matrix interphase, which in turn significantly affects the performance of the resulting composites^3^. Nanoparticle-reinforced polymer composites demonstrate significant improvements in mechanical, thermal, and barrier properties at low filler concentrations, exceeding the performance of pure polymer matrix^4–6^. A small modification in the matrix system and addition of low wt.% nanoscale fillers into composites leads to enhancements in mechanical behavior, dynamic mechanical, thermal behaviors, T_g_, water-resistance rate, thermal conductivity, coefficient of thermal expansion, load transfer efficiency and even dispersion of the carbon fabric-reinforced epoxy (CF-Ep) composites^7,8^. The smallest wt.% NC filled CF-Ep composites gave the better mechanical behavior and higher storage modulus and T_g_ values compared to higher wt.% NC composites. The hierarchy or ranking of performance of the unfilled and 2.5%, 5% and 10% NC filled CF-Ep composites varied in each DMA property analysis such as SM, LM, Tanδ and T_g_^9^. The presence of nano and micro fillers significantly affected the mechanical behavior and wear resistance, primarily due to the stability of transfer film formed on the counter surface^10^. Carbon fiber–reinforced epoxy composites (CF-EPs) are widely recognized for their high strength-to-weight ratio, excellent stiffness, and thermal stability, offering superior performance compared to glass or natural fiber–reinforced composites and thermoplastic matrices^11,12^. The dispersion of nanoparticles, such as halloysite nanotubes (HNTs), within the epoxy matrix plays a critical role in enhancing mechanical properties by improving interfacial bonding, stress transfer, and crack deflection, as discussed in^13^. The novelty of this study lies in optimizing the HNT content in carbon fiber–epoxy composites through ultrasonication-assisted dispersion and systematically analyzing the wear, mechanical, and fracture behavior under different loading conditions, establishing a direct relationship between nanofiller dispersion and composite performance.

The Halloysite nanotubes (HNTs) are the aluminosilicate nanotubes Al_2_Si_2_O_5_(OH)_4_·2H_2_O which are cheaper than other nanofillers, especially CNTs^14–17^. In the polymer matrix, the HNTs effective dispersion improves various characteristic features, but it is a very tedious process because of its agglomeration behavior^18–20^. The HNTs have considerable quantity of water between the AlO_6_ and SiO_4_ structure^21–23^. The behavior of HNTs filled polymer composites and its structure depends largely on dispersion ability, concentration and temperature. Ultrasonication is widely used method to mitigate the effect of agglomeration, thereby uniform mixing of HNTs powder in the resin system results in enhancement of mechanical and tribological behavior^24,25^.

CF-Ep composites possess self-lubricating properties and have been widely utilized in bearings functioning without liquid lubricants. During the bearing sliding tests, the generation and compression of wear debris were the primary factors influencing friction, wear, and lubrication. For the reduction of friction, wear, etc., the wear debris compaction should be reduced which is possibly attained by generating several micro-grooves on the test surface^26^. Different reinforced materials of different states were systematically compared for tribological behavior. The fiber reinforcement with filler particle addition effect on Ep-matrix composites was also tested for sliding conditions. It was found that COF of CF composites was reduced by 35% when compared to GF composites. This decrease is considered to be due to the lubricating effect of CFs as opposed to the abrasive nature of GFs. At the lowest PV stage (0.25 MPa m/s and 0.50 MPa m/s), the CF composites showed great SWR, while at the three roughest PV stage (0.75 MPa m/s, 1.00 MPa m/s and 1.50 MPa m/s), the glass fiber composites exhibited relatively steady tribological performance regardless of decomposition and the development of large-scale cracks^27^. The CF-Ep composites showed better mechanical behavior when tested for tensile, wear and flexure^28^. The incorporation of 0.5 wt% CNTs, as reported by Xian et al.^29^, enhanced the self-healing performance of epoxy composites by improving thermal conductivity for efficient PCL activation, reinforcing the matrix for better mechanical strength, and promoting stable interaction and dispersion within the epoxy–PCL system. Additionally, according to a study by Xian et al.^30^, adding 7.5 wt.% PA6 to carbon fiber-reinforced epoxy resin greatly increased its fracture toughness by 198.6%, tensile strength by 34.0%, and elongation at break by 77.3%. This showed superior mechanical improvement and thermodynamic compatibility, but hygrothermal aging at 60 °C for 120 days resulted in notable property degradation because of resin hydrolysis and filler-matrix interface debonding.

Good correlation / similarity between the WVL and various mechanical behaviors seem to be obtained in the unfilled and NC, EVA filled LDPE composites with and without the compatabilizing blends. SEM of unfilled EVA at low abrading distance precisely exhibits wear patterns following the direction of abrasive flow. Here, the hard sand abrasives penetrate inside the sample surface thereby undergoing micro-ploughing process and removes material from the surface^31^. POD analysis revealed that interface material significantly affects wear. CF-Ep composite wear was primarily due to fiber fracture and micro-cracking. In contrast, filler-filled CF-Ep composite wear was mainly micro-ploughing, resulting in superior wear behavior^32^.

A good number of researchers have analyzed the wear resistance diminishes by the addition of fillers and fibers and Ravikumar et al. have showed both increased the wear^33^. Material removal is lesser in nano-additives since these have sizes similar to surrounding polymer chain segments. Despite these positive effects, still, segments have few critical problems that are unanswered, and this shows that nano-fillers don’t always guarantee the improvement of friction and wear behavior. There are various material behaviors other than scratch penetration depth, SWR, etc. which are the sole indicators to compare, rank and analyze composite behavior^34^.

It has also been proved application-wise that HNT filler enhances behavior that help them to be used in biological applications compared to mechanical, thermal and tribological applications. Lesser exposure and importance are given to its usage in most engineering applications. There is comparatively less research on the usage and applications of HNT for balanced boost in mechano-thermal and tribological behavior apart from the present biological applications. Also, very little research has been seen on surface weathered HNT powder-filled CF-Ep composites considering 2 × 2 twill weave CF structure. This research examines the effect of HNT powder on the mechanical, tribological, and thermal properties of CF-Ep composites relevant to automotive, tribology and other applications.

## Experimentation

### Materials

The matrix was made of Huntsman’s Araldite LY1564 epoxy resin, which was hardened using Aradur 22,962 (CF Composites, Delhi, India). The T800 bidirectional carbon fiber (Toray, 2 × 2 twill weave), which was also obtained from CF Composites, Delhi, India, served as reinforcement. For the mold, Huntsman’s QZ-13 grade releasing agent was utilized. Sigma Aldrich, India) provided the HNTs, a naturally occurring aluminosilicate mineral with primarily tubular structures and particle sizes less than 10 µm. Natural aluminosilicate minerals called as HNTs resemble kaolin chemically but primarily have a tubular structure as a result of surface weathering or hydrothermal alteration^35,36^. Table 1 lists the characteristics of HNT fillers. Grade III AFS 60 quartz sand (Tamilnadu Minerals Limited, Chennai) was sieved to isolate particles of 212 µm size, which were subsequently used for TBA wear testing and ANOVA analysis.

**Table 1:** Physical and Mechanical Properties of HNTs.

Property	Structure/value/unit
Crystal structure	Hexagonal
Purity	98%
Melting point (°C)	3000
Form	Powder
Appearance	Colorless crystals
Density	2.29 gm L^−1^ at 25 °C (lit.)
Young’s modulus (MPa)	20–100
Solubility in water	Insoluble
Tensile strength, flexural strength, stiffness	Significantly enhances
Thermal stability	Excellent at higher temperatures
Crystallization	Promote crystallization in polymers, affecting the spherulite size and growth rate
Surface chemistry	Modified to enhance compatibility with different matrices
Surface area	Large
Aspect ratio	High
Lengths	1–15 µm
Inner diameter	10 and 30 nm
Outer diameters	50–70 nm

### Ultrasonication and fabrication

**Fig. 1:**
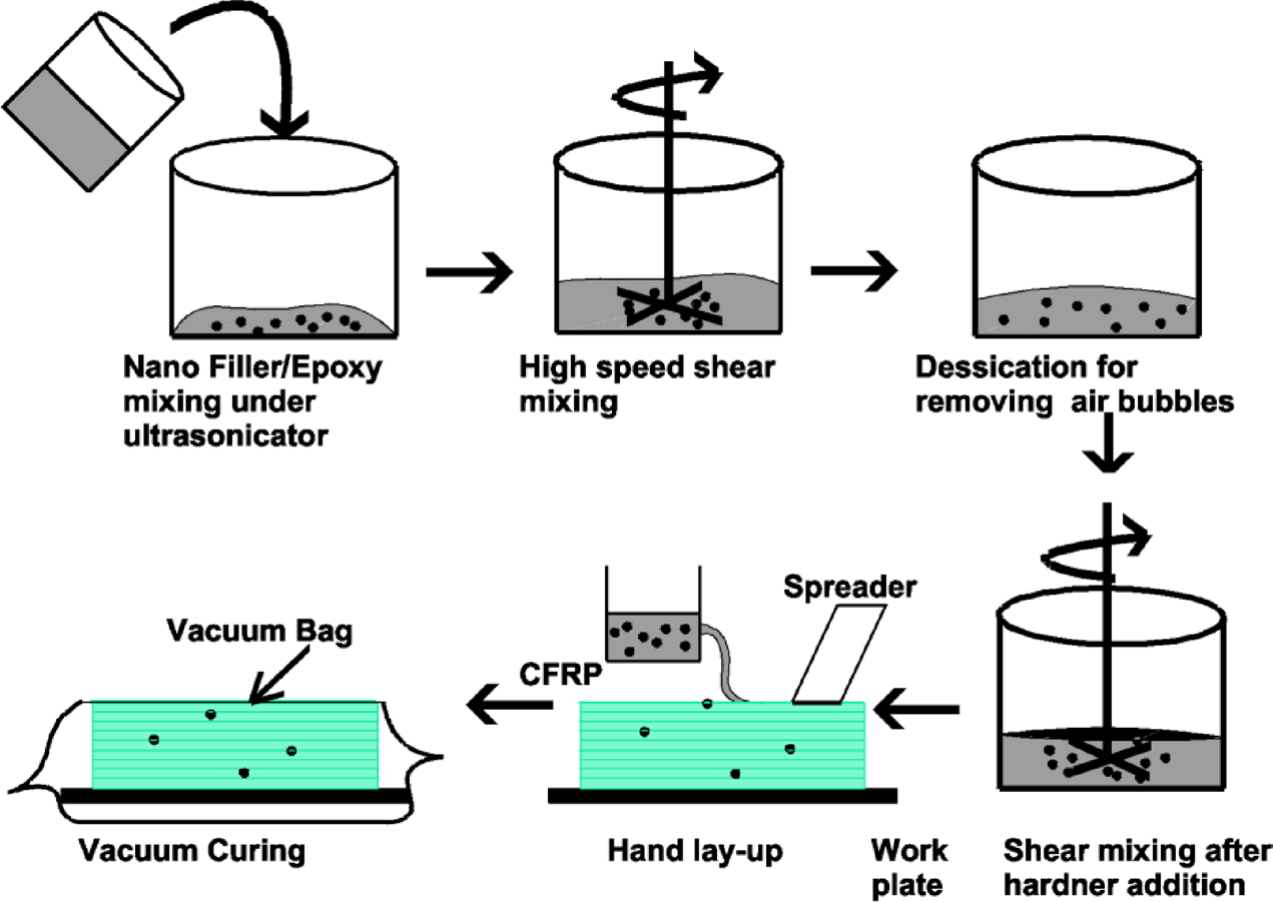
Ultrasonication process for dispersion of HNT in CF-Ep composites.

**Fig. 2:**
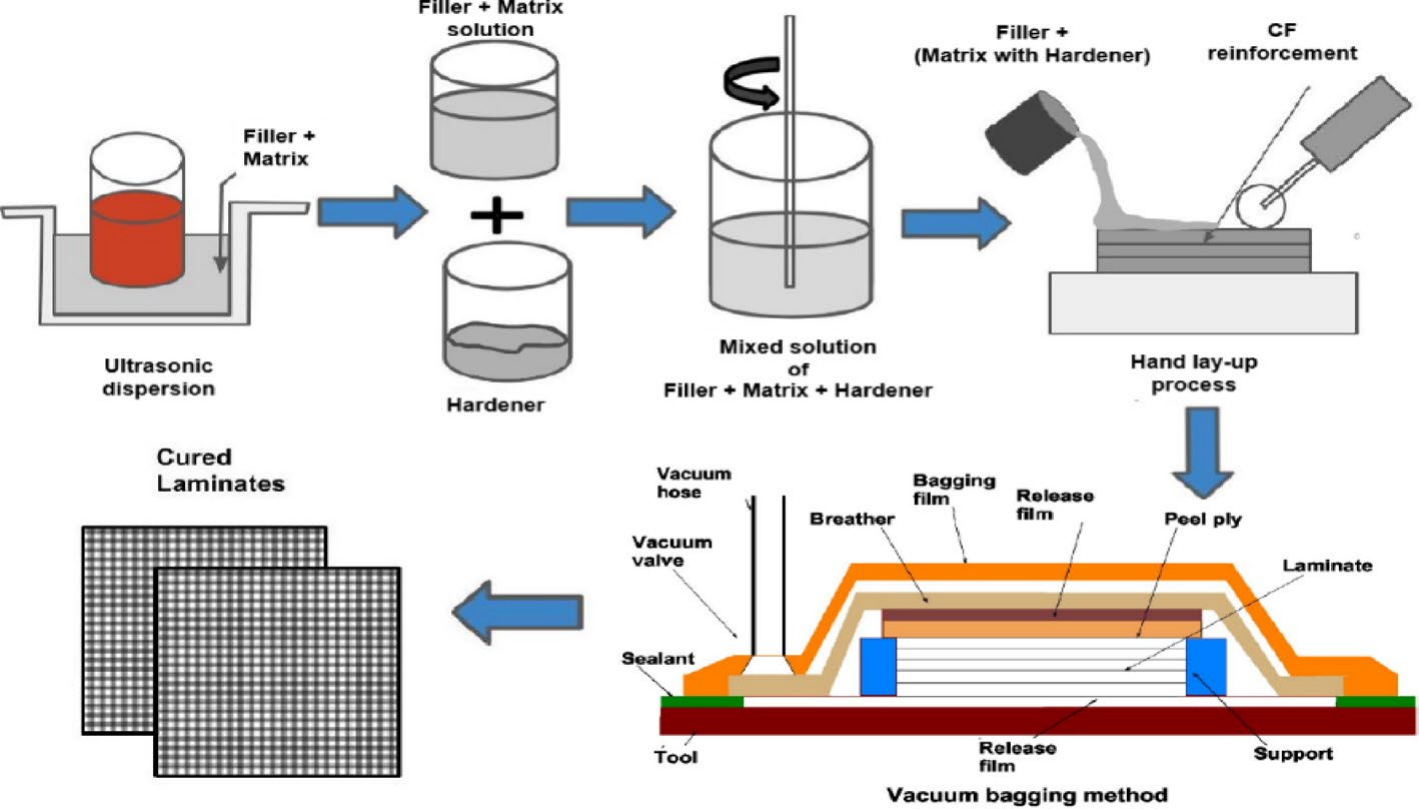
Processability of HNT-filled composites.

The dispersion of HNTs into the epoxy (Ep) matrix via ultrasonication (Fig. 1) and the subsequent fabrication of HNT-filled carbon fiber–epoxy (H-CF-Ep) composites (Fig. 2) were performed according to the methodology detailed in a previous study^37^. To ensure uniform dispersion of particulate fillers within the epoxy matrix, high-intensity ultrasonic waves were applied to the filler–epoxy mixture using a probe sonicator (Johnson Plastosonic) for 40 min at a frequency of 20 kHz, with a pulsed cycle of 5 s on and 5 s off. The ultrasonication duration was selected considering the influence of both the matrix material and the filler type. Ultrasonication was carried out in a water bath to prevent thermal degradation of the epoxy, ensuring better filler dispersion and reduced agglomeration compared to manual mixing.

To facilitate comprehension of the fabrication section of this study, the composites are displayed in Table 2 and Fig. 2, respectively, along with the fabricated laminates and their- composition and the overall processability of the H-CF-Ep. Fabrication was performed using the vacuum bagging method after the hand lay-up procedure. Vacuum bagging is performed by enclosing the laminate in a sealed polyethylene bag and applying vacuum pressure (up to 1 atm or 101 kPa) to ensure consolidation. A release film is placed over the laminate, followed by a peel ply or absorbent polyester cloth to remove excess resin during curing. The process improves fiber volume fraction and enhances interlayer bonding. For superior laminate quality, vacuum curing is often combined with elevated temperature. The epoxy (with filler) and hardener were mixed in a weight ratio of 100:25. The mixture was then subjected to hydraulic pressing at approximately 0.5 MPa and allowed to cure naturally at room temperature (RT) for 24 h. The laminates were cured between (90 °C and 100 °C) for 60 min within an oven. The laminates were prepared to the (300 mm × 215 mm × 4 mm) size. A (60:40) reinforcement: matrix ratio was maintained.

**Table 2:** Fabricated laminates and their composition (wt.%).

Composite (Designation)	CF (wt.%)	(Ep + HNT)
CF + Ep (CF-Ep)	60	(40 + 0.00)
CF + Ep + HNT0.75% (H0.75%)	60	(39.25 + 0.75)
CF + Ep + HNT1.75% (H1.75%)	60	(38.25 + 1.75)
CF + Ep + HNT2.75% (H2.75%)	60	(37.25 + 2.75)

HNTs offer better dispersibility in polymer matrices than many other nanofillers due to their rod-like shape and lower contact area. Their surface hydroxyl groups enable functionalization with silane agents, improving both dispersion and interfacial bonding.

### Characterization

#### Mechanical tests

Tensile testing was carried out in accordance with ASTM D638 using a universal testing machine (UTM) equipped with a 100 kN load cell and operated at a crosshead speed of 10 mm/min. Flexural and interlaminar shear strength (ILSS) tests were performed using the three-point bending method, following ASTM D790 and ASTM D2344 standards, with crosshead speeds of 5 mm/min and 2.5 mm/min, respectively. Hardness measurements were obtained using a Shore durometer, as specified in ASTM D2240.

#### Differential scanning calorimetry

Evaluation of curing behavior of the composite was investigated using Differential Scanning Calorimetry (DSC) performed by “DSC 6000 Pyris 6 DSC version 11.0.0.0449” instrument with the heating range of (30–440 °C) at the heating rate 10 °C/min, nitrogen atmosphere as per the ASTM D3418 standard.

#### Fourier transform infrared spectroscopy

Chemical characterization of the fabricated unfilled and HNT-filled CF/Ep composites was performed using Fourier Transform Infrared (FTIR) spectroscopy, following the ASTM D5477 standard. This analysis was conducted to assess filler dispersion and curing behavior. FTIR spectra were recorded in transmission mode at room temperature using a PerkinElmer Spectrum system (Version 10.03.09) across the wavenumber range of 4000–450 cm⁻^1^.

#### Dynamic mechanical analysis test

Dynamic Mechanical Analysis (DMA) tests were conducted on composite samples (30 mm × 10 mm × 2 mm) using a TA Instruments DMA Q800 V7.4 Build 126 instrument. All the dynamic tests were conducted using a 3-point bending fixture and ASTM D7028 standard. The samples were subjected to a 1 Hz oscillating force while heating from – 50 °C to 250 °C at 5 °C/min. Storage modulus (E′), loss modulus (E′′), and damping factor (tan δ) were measured to evaluate the material’s viscoelastic behavior, stiffness, and energy dissipation. The glass transition temperature (T_g_) and other viscoelastic properties were determined from the data. The T_g_ was known from the peak in the tanδ curve.

#### Abrasive and dry sliding wear tests

Tribological performance was investigated under two distinct wear conditions, namely three-body abrasion (TBA) and dry sliding friction wear (DSFW). TBA tests were conducted using a dry sand rubber wheel abrasion tester (Magnum Engineer’s, Bengaluru, India) with silica sand (212 µm) abrasives at a constant load of 30 N and sliding velocity of 2.38 m/s, with abrading distances from 250 to 1000 m. In the DSFW test, a pin-on-disc tribometer (Magnum Engineer’s, Bengaluru, India) was used under a load of 60 N and a sliding speed of 3 m/s, with sliding distances ranging from 1000 to 3000 m, in accordance with ASTM G99.

The ASTM G65 standard procedure was used for performing the dry sand abrasive wear tests. Quartz sand of particle size 212 µm was taken as abrasive sand, which was fed between the rotating rubber wheel and the test sample. The tests are conducted at a speed of 200 revolutions per minute and at a fixed sliding velocity of 2.38 m/s. The abrasive sand was fed at a controlled rate of 255 ± 5 g/min. Prior to testing, the specimens were cleaned with acetone, thoroughly dried, and their initial weight was recorded using a digital balance with 0.1 mg precision. The rubber wheel employed had a diameter of 228 mm. The tests were carried out for four levels of normal loads (10N, 20N, 30N, 40N), abrading distances (250 m, 500 m, 750 m, 1000 m) and filler weight% (0%, 0.75%, 1.75%, 2.75%). After the set test duration, removal of the specimen was done, cleaned thoroughly and weighed again (final weight). The weight difference before and after abrasive wear tests were found and are called the weight loss. Wear volume loss (ΔV) in (mm^3^ × 10^3^) was calculated by the following Eq. 1,

1 $${\text{Wear volume loss}}, \, \Delta {\text{V = }}\frac{{\Delta {\text{W}}}}{\rho }{\text{where}}, \, \Delta {\text{W}} = {\text{change in weight }}\left( {\text{g}} \right), \, \rho \, = {\text{ Density }}\left( {{\text{g}}/{\text{cm}}^{{3}} } \right)$$

#### Optimization technique

Optimization aims to identify the most effective combination of parameters that yield the best performance outcome from multiple variables and constraints. In this study, the Taguchi method was applied as a statistical approach to evaluate the influence of multiple process parameters and their interactions on wear performance. This method reduces the number of experimental trials, thereby improving efficiency in terms of time, effort, and cost. An experimental plan was developed using the Taguchi approach with a standard L16 orthogonal array, involving three control factors such as load (L), abrading distance (D), and HNT filler content (F) each at four levels. The tests were conducted on 212-HNT epoxy composites under dry sand three-body abrasion conditions. The experimental design and levels of the variables are presented in Table 3. The resulting wear volume loss (WVL) data were analyzed using regression modeling, with the response equation expressed as:

2 $$Y = b_{0} + b_{{1}} X_{{1}} + b_{{2}} X_{{2}} + b_{{3}} X_{{3}} + b_{{{12}}} X_{{1}} X_{{2}} + b_{{{13}}} X_{{1}} X_{{3}} + b_{{{23}}} X_{{2}} X_{{3}} + b_{{{123}}} X_{{1}} X_{{2}} X_{{3}}$$

where Y is the wear volume loss, b_0_ is the response to the variables, where*b*_1_, *b*_2,_ *b*_3_ are the coefficients for each variable (load, abrading distance and filler wt.% respectively, linear constants) within the selected level of each variable. Also coefficients *b*_12_, *b*_13_,* b*_23_ and *b*_123_ are the interaction constant terms. *X*_1,_ *X*_2_ & *X*_3_ represent the factors, load, abrading distance and filler wt.% respectively. The positive and negative values of the coefficients indicate the increase and decrease of WVL respectively with an increase and decrease in the respective parameters.

**Table 3:** Experimental parameters for 212-HNT composite evaluation.

Control factors		Levels			
		I	II	III	IV
212-HNT composites	Load (N)	10	20	30	40
	Abrading distance (m)	250	500	750	1000
	Filler (%)	0	0.75	1.75	2.75

Analysis of Variance (ANOVA) was performed to determine the statistical significance and percentage contribution of each factor to the response. Signal-to-noise (S/N) ratio analysis, based on the “smaller-the-better” criterion, was used to evaluate wear resistance. All statistical evaluations were conducted at a 95% confidence level using MINITAB 17 software.

#### Microstructure study

Scanning Electron Microscopy (SEM) micrographs (JEOL JSM-6390) operating at an accelerating voltage of 10–20 kV were used to characterize the morphology of tensile fractured samples and worn surfaces.

## Results and discussion

### Tensile properties

**Fig. 3:**
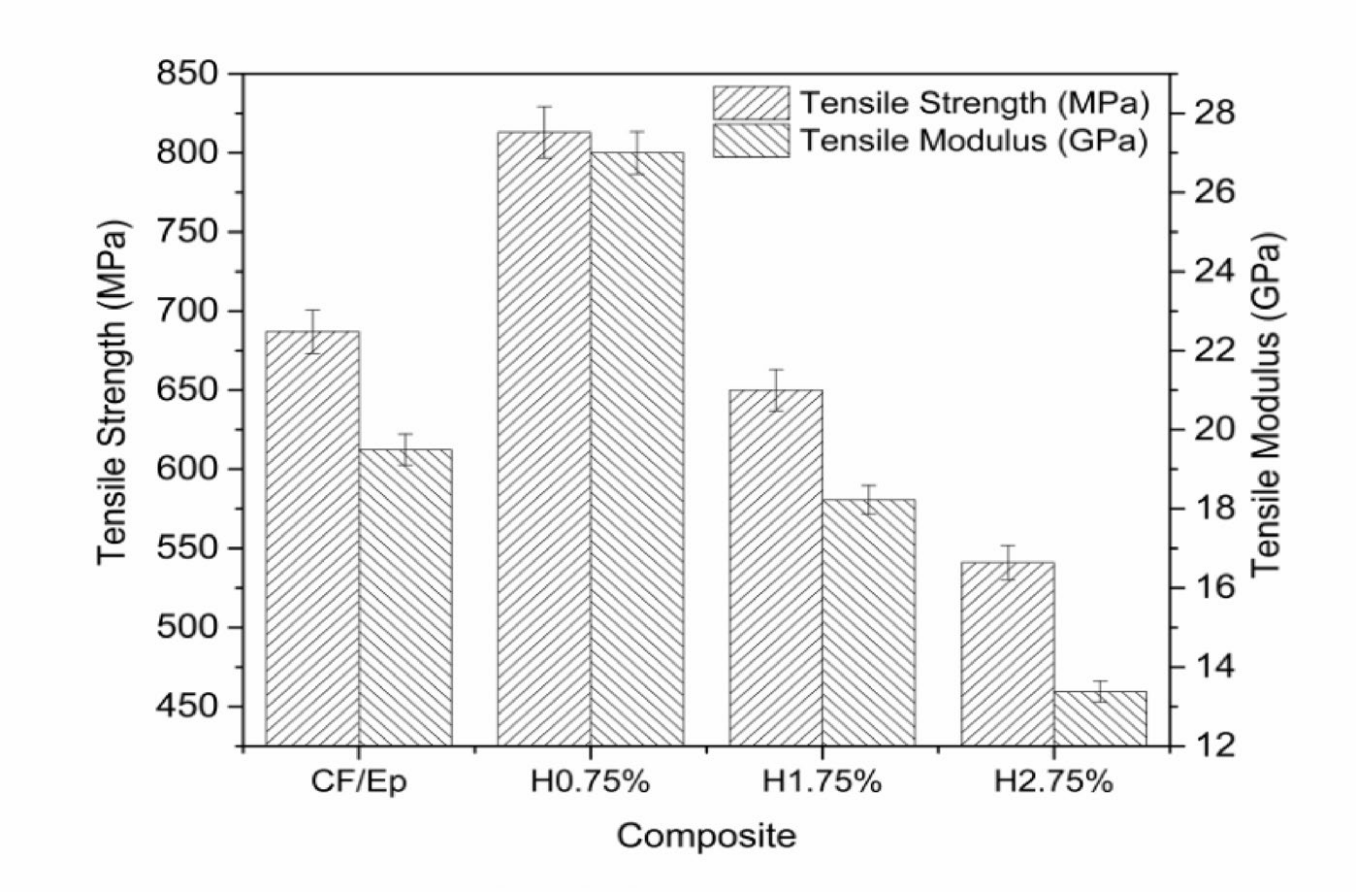
Tensile strength of unfilled and HNT-reinforced composites.

The mechanical properties of the composites, as shown in Fig. 3, varied significantly with changes in additive concentration. The control composite (CF-Ep) exhibited a tensile strength of 680 MPa and a tensile modulus of 19 GPa. Incorporating 0.75 wt.% HNT notably improved these properties, increasing tensile strength to 820 MPa and modulus to 27 GPa, indicating optimal reinforcement. However, higher additive contents led to a decline in performance: 1.75 wt.% HNT reduced the tensile strength to 650 MPa and modulus to 20 GPa, while 2.75 wt.% resulted in further deterioration, with values dropping to 540 MPa and 16 GPa, respectively. The improvement at 0.75 wt.% is attributed to strong adhesive bonding at the fiber-matrix interface, which enhanced stress transfer and minimized structural defects. In contrast, the unfilled CF-Ep composite showed weaker bonding and more defects such as the formation of voids and the occurrence of fiber pull-out. The addition of HNT mitigated these issues, with 0.75 wt.% HNT yielding the best mechanical performance.

The tensile strength of H-CF-Ep composites was improved to 810 MPa (for H0.75% composites) from 687 MPa (for CF-Ep composites), nearly 17.90% higher. The tensile moduli of H0.75% composites were enhanced by 37% from unfilled composites (19.6 GPa). The HNTs improved tensile behavior by facilitating uniform mixing with the Ep, enabling effective stress transfer from the Ep to the CFs. A good dispersion reduces the void content thereby increases the available surface for uniform distribution of the applied load^38,39^.

**Fig. 4:**
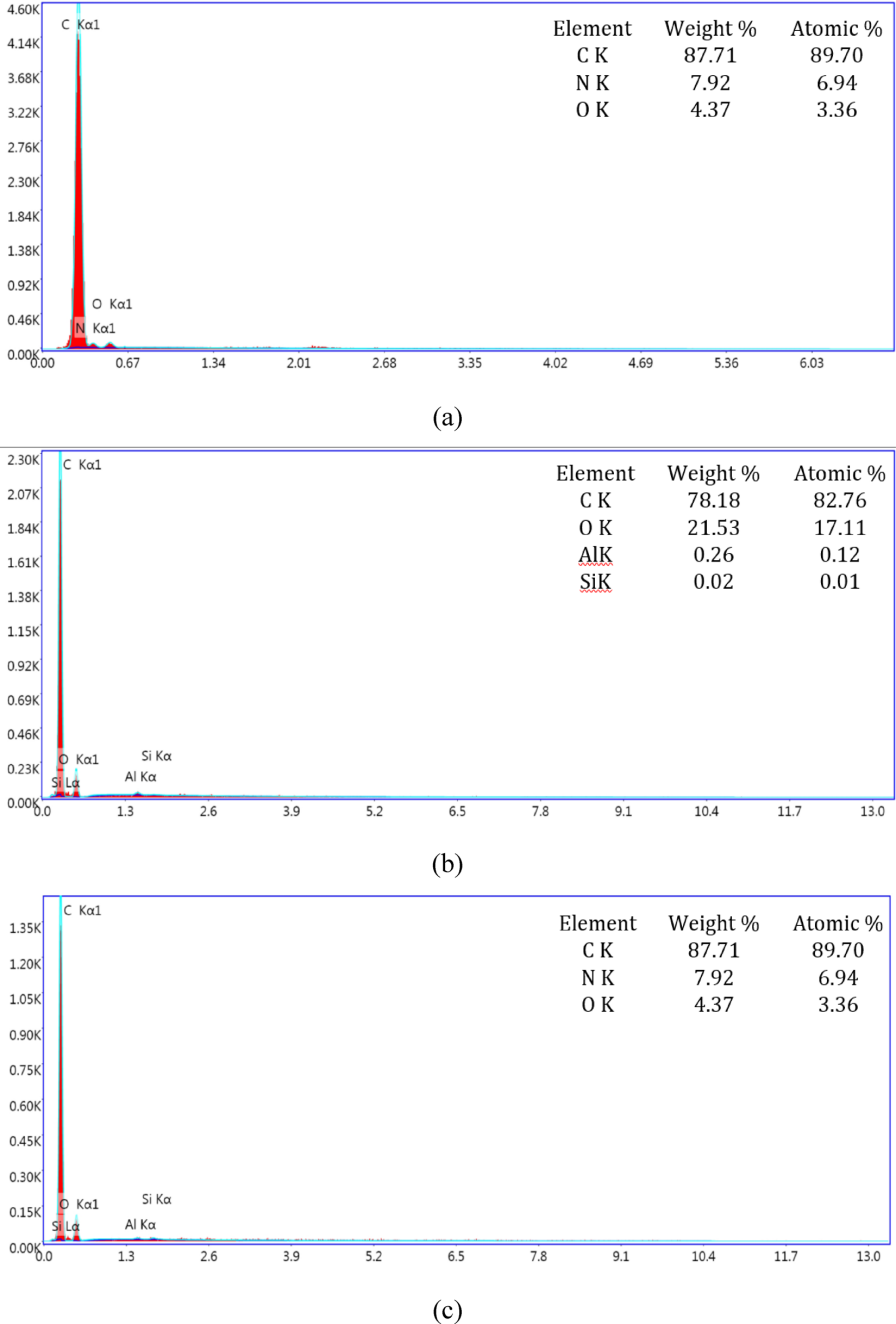

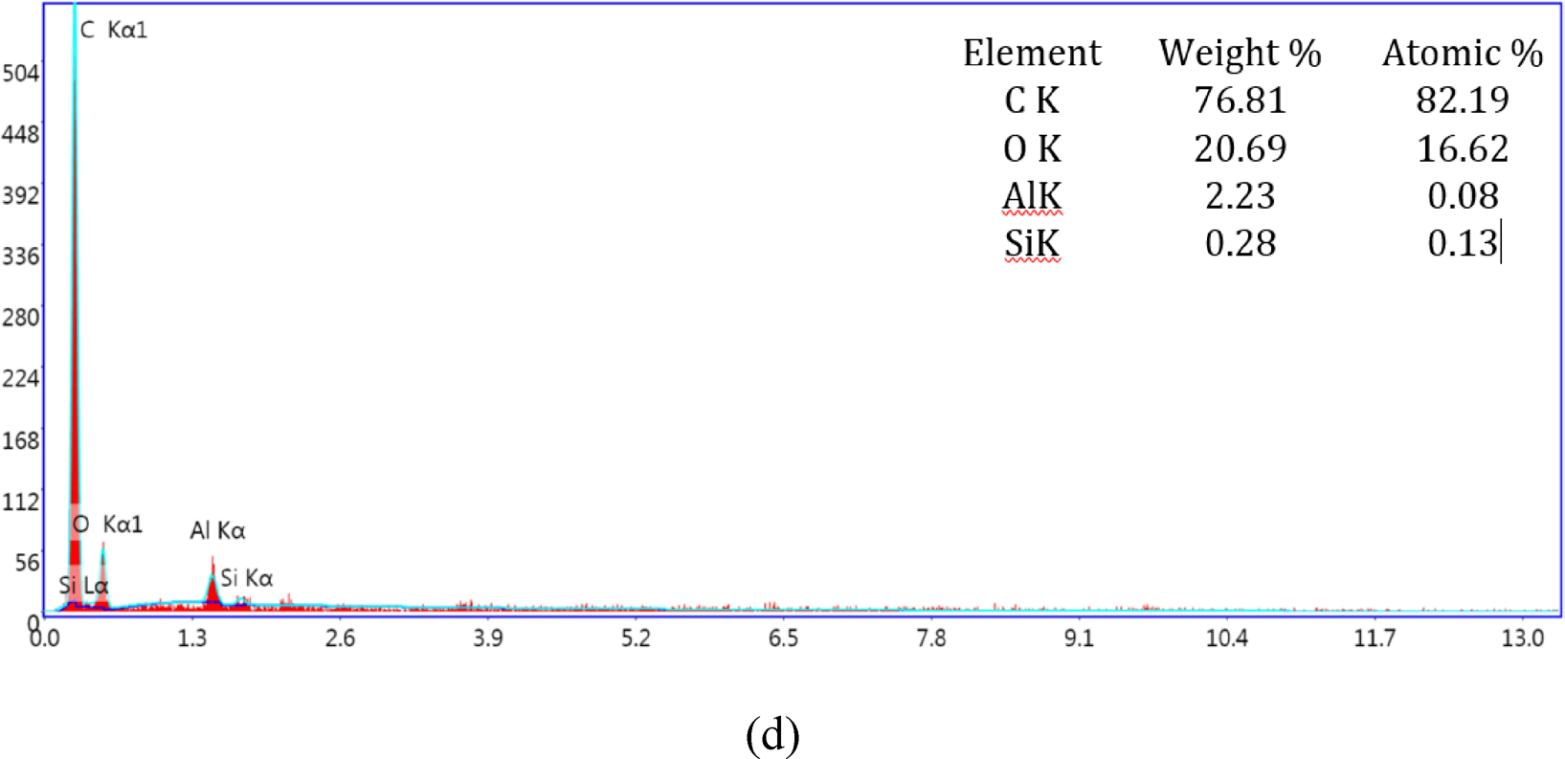
EDAX of unfilled and HNT filled CF-Ep composites: (**a**) CF-Ep, (**b**) H0.75%, (**c**) H1.75%, (**d**) H2.75%

Figure 4 shows the EDAX (Energy Dispersive X-ray Analysis) of unfilled and HNT filled CF-Ep composites. The presence of CF-Ep is identified using characteristic peaks of carbon and oxygen, as well as any other elements present in the epoxy resin. Carbon (C) and oxygen (O) are typically the most prominent peaks in CF-Ep composite (Fig. 4a) advocates the presence of CF-Ep in composites. Similarly, the HNT with chemical formula (Al_2_Si_2_O_5_ (OH)_4_nH_2_O), has the most prominent peaks of Aluminium and Silicon and even their weight percentages are high indicating the presence of HNT in CF-Ep composites. Figure 4 shows the total wt.% of Al and Si content combined of H0.75, H1.75 and H2.75 composites are 0.28%, 0.36 and 2.51% respectively, i.e., as per EDAX, all the H-CF-Ep composites are within their filled wt.% limit of 0.75%, 1.75% and 2.75% respectively. And also, it confirms the even distribution of HNT in CF-Ep composites within the ultrasonication process limit. The incorporation of HNTs enhanced the properties of CF-Ep composites compared to the unfilled, primarily due to restricted chain mobility, decreased epoxy matrix deformability, and the ordered exfoliation of polymer chains within the interstitial spaces of the HNT^24^. The HNT dispersion in Ep matrix enhanced the static adhesive strength and interfacial stiffness of CF-Ep composites by transferring the elastic deformation to a large extent. In H-CF-Ep composites, the HNT further addition into Ep matrix showed decreased tensile behavior due to HNT aggregation across the Ep system matrix leading to stress concentration. Hence concluded that there will be optimum wt.% of HNT that disperses uniformly in Ep system, beyond that there will be decrement of mechanical behavior. The agglomerates behave like stress enhancers, lowering the breaking stress required for the sample failure. Singh et al. showed that the unfilled glass-polyester composites showed higher tensile strength whereas the filler filled bi-directional glass-polyester composites presented lower strength due to the increment of void fraction in composites^40^. Similarly, here the H1.75% and H2.75% composites presented lower tensile strength and H0.75% presented higher strength than unfilled ones. The measured tensile elongation before failure was 9.55 mm for unfilled CF-Ep, increased to 10.47 mm for H0.75%, and then decreased to 9.25 mm and 9.12 mm for H1.75% and H2.75% composites, respectively. The elongation values followed a similar trend to the tensile behavior, where higher strength and modulus corresponded to greater elongation. The HNTs acted as a bridge, forming chemical bonds between the CFs and the Ep matrix. The HNTs formed an interfacial chemical integration between the CF and HNT-Ep matrix in the H-CF-Ep composites. The impact of different HNT contents on mechanical properties has been explained by additional analysis. HNTs are evenly distributed at 0.75 wt.%, improving stress transmission and interfacial bonding without leading to agglomeration. Particle clustering brought on by higher loadings lowers mechanical performance and reinforcing efficiency.

### Flexural properties

**Fig. 5:**
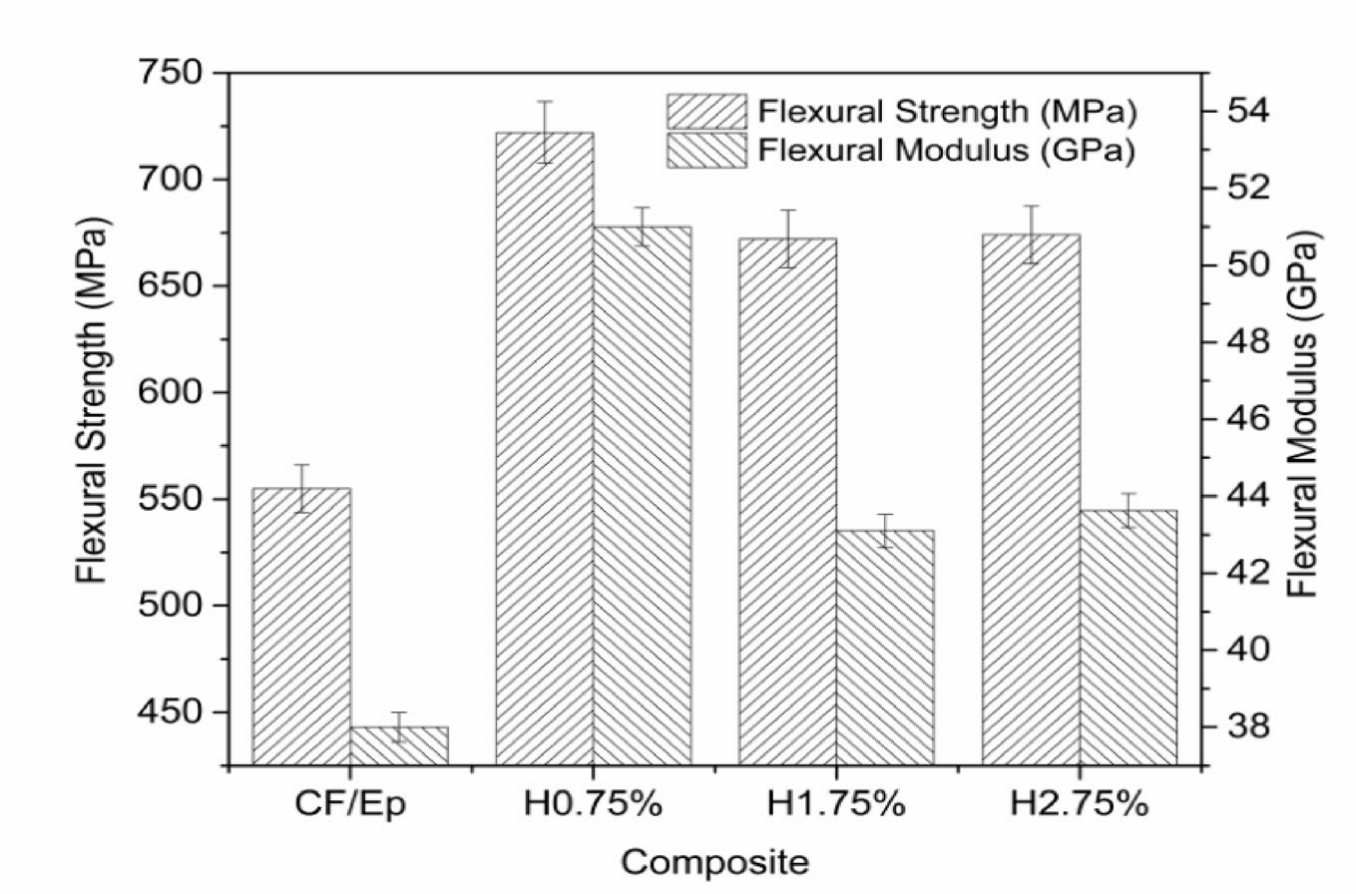
Flexural strength of unfilled and HNT-filled composites.

The flexural strengths of H0.75%, H1.75%, H2.75%, and unfilled CF-Ep composites are illustrated in Fig. 5. The test results revealed that H0.75% composites exhibited the highest flexural behavior, outperforming H1.75%, H2.75%, and unfilled CF-Ep composites^41^. The addition of HNTs enhanced flexural behavior up to a certain point, with H0.75% showing optimal performance. However, further increases in HNT content led to decreased performance due to defects such as micro-voids, cracks, and poor bonding^24^. The HNT promoted a plasticizing effect, resulting in higher toughness and effective stress transfer between the CF and Ep matrix^42^. This was attributed to the strong interface bond and excellent chemical bonding at the CF-Ep assembly, ultimately leading to enhanced flexural behavior^43^. The incorporation of 0.75 wt.% HNT (H0.75%) resulted in optimal flexural performance, with a 29% increase in flexural strength and 34% increase in flexural modulus compared to unfilled CF-Ep composites. However, further increases in HNT content led to reduced flexural properties.

The flexural strength and modulus enhancements were accompanied by increased deflection values, with H0.75% composites exhibiting the highest deflection of 6.99 mm. The observed trend suggests a direct correlation between flexural strength, modulus, and deflection, where higher strength and modulus correspond to greater deflection. The impact of different HNT contents on flexural strength has been explained by additional analysis. HNTs are evenly distributed at 0.75 wt.%, improving stress transmission and interfacial bonding without leading to agglomeration. Particle clustering brought on by higher loadings lowers mechanical performance and reinforcing efficiency.

### Interlaminar shear strength

**Fig. 6:**
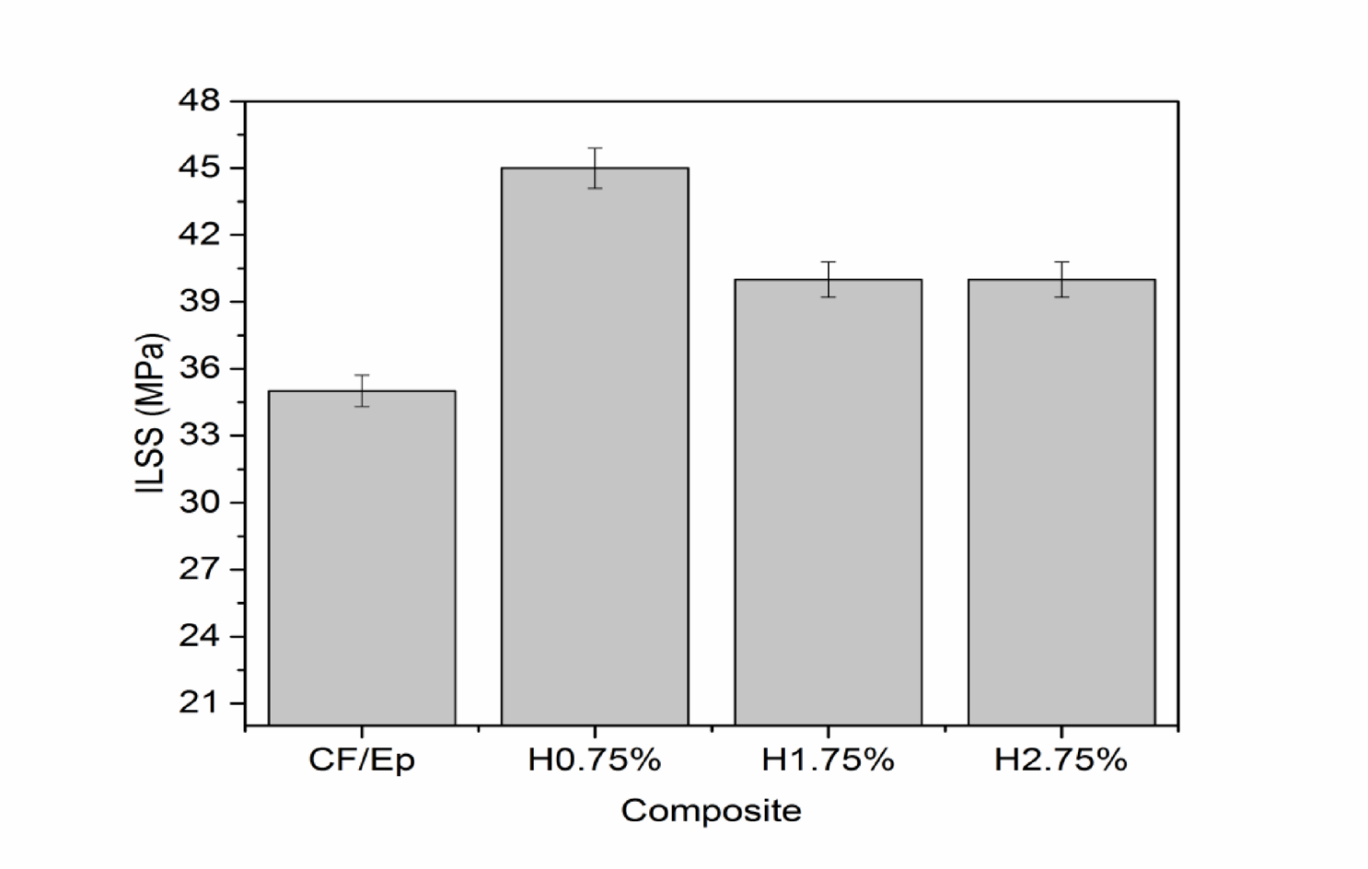
ILSS of unfilled and HNT-filled composites.

Figure 6 shows the interlaminar shear strength (ILSS) of the composites. The incorporation of HNT led to significant enhancements in ILSS, with H0.75%, H1.75%, and H2.75% composites exhibiting increases of 28%, 14%, and 14%, respectively, relative to unfilled CF-Ep composites. This enhancement was ascribed to the homogenous distribution of HNTs within the epoxy matrix, which enhanced fiber bonding and load transfer. Consistent findings have been reported by Ravichandran et al.^24^ and Qin et al.^42^, where Inclusion of nano-fillers resulted in an increased ILSS, attributed to enhanced interfacial bonding and a reduced void fraction. The impact of different HNT contents on ILSS has been explained by additional analysis. HNTs are evenly distributed at 0.75 wt.%, improving stress transmission and interfacial bonding without leading to agglomeration. Particle clustering brought on by higher loadings lowers mechanical performance and reinforcing efficiency.

### Hardness

**Fig. 7:**
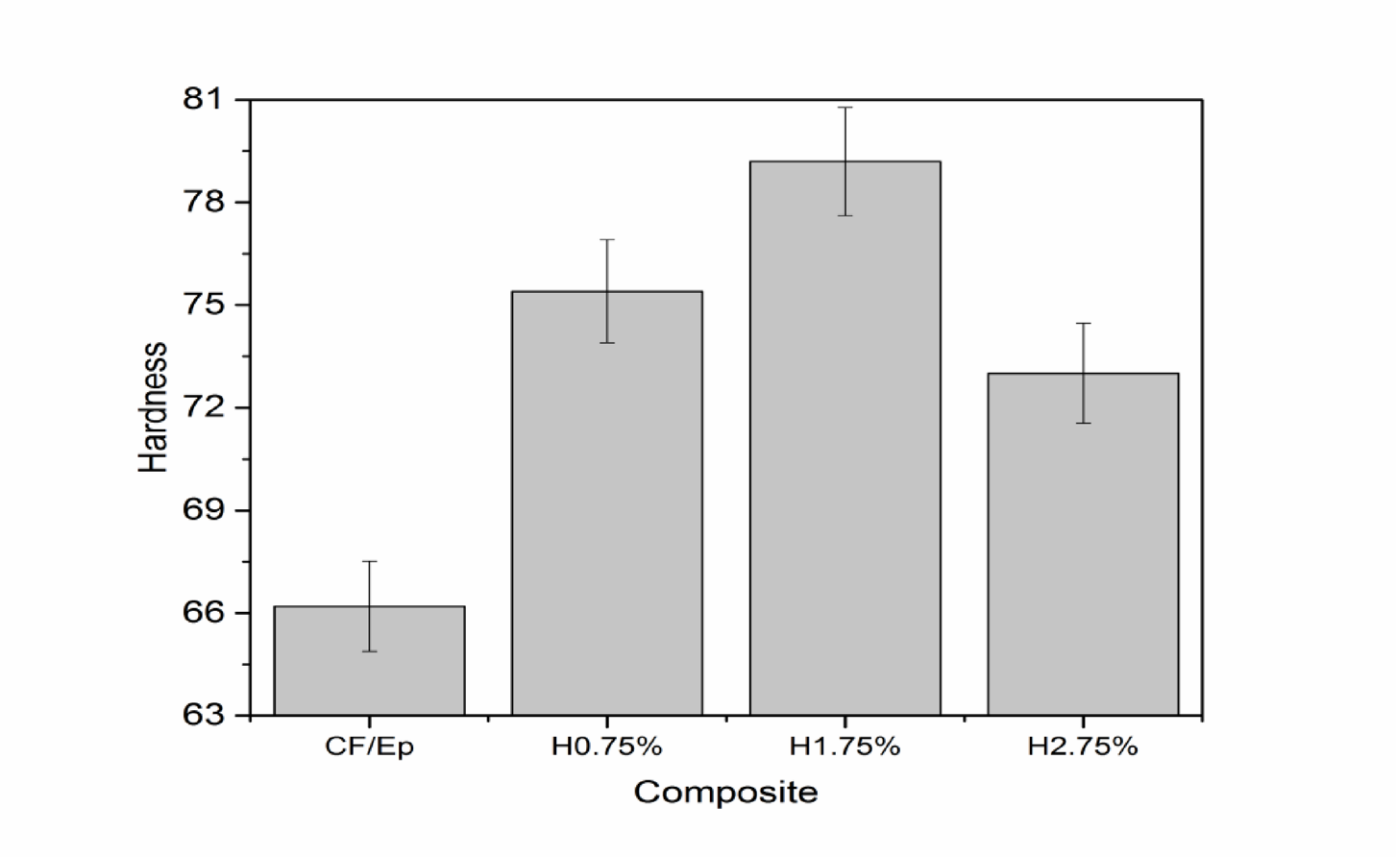
Hardness of unfilled and HNT-filled composites.

The Shore D hardness values of the unfilled and HNT-filled CF-epoxy composites are shown in Fig. 7. Since HNTs have a rigid structure and are evenly distributed throughout the epoxy matrix, their addition resulted in a discernible increase in hardness when compared to the unfilled composite^24^. The stiffer composite surface produced by this distribution increases resistance to indentation and decreases matrix deformability. Due to optimal filler dispersion, appropriate curing, and effective stress transfer from the matrix to the filler, the H1.75% composite had the highest recorded hardness value, with Shore D hardness rising from 66.2 (CF-Ep) to roughly 79^39^. This demonstrates improved stiffness and interfacial bonding. In spite of the higher filler content, the H2.75% composite showed a decrease in hardness. Agglomeration of the nanoparticles, which increases with increasing loadings, can account for this reduction. The excess HNTs are not completely distributed at 2.75 wt.% and start to cluster into tiny clusters inside the epoxy matrix. These aggregates compromise the material’s homogeneity and lower its load-bearing capacity under indentation because they function as stress concentrators rather than reinforcement points. Moreover, inappropriate matrix cross-linking during curing may be impeded by such clustering, decreasing interfacial strength and adversely influencing overall hardness. Therefore, even though adding HNT usually increases hardness, the ideal loading is 1.75 wt.%; above this, performance suffers from dispersion restrictions and microstructural flaws.

### Tensile fractured morphology

**Fig. 8:**
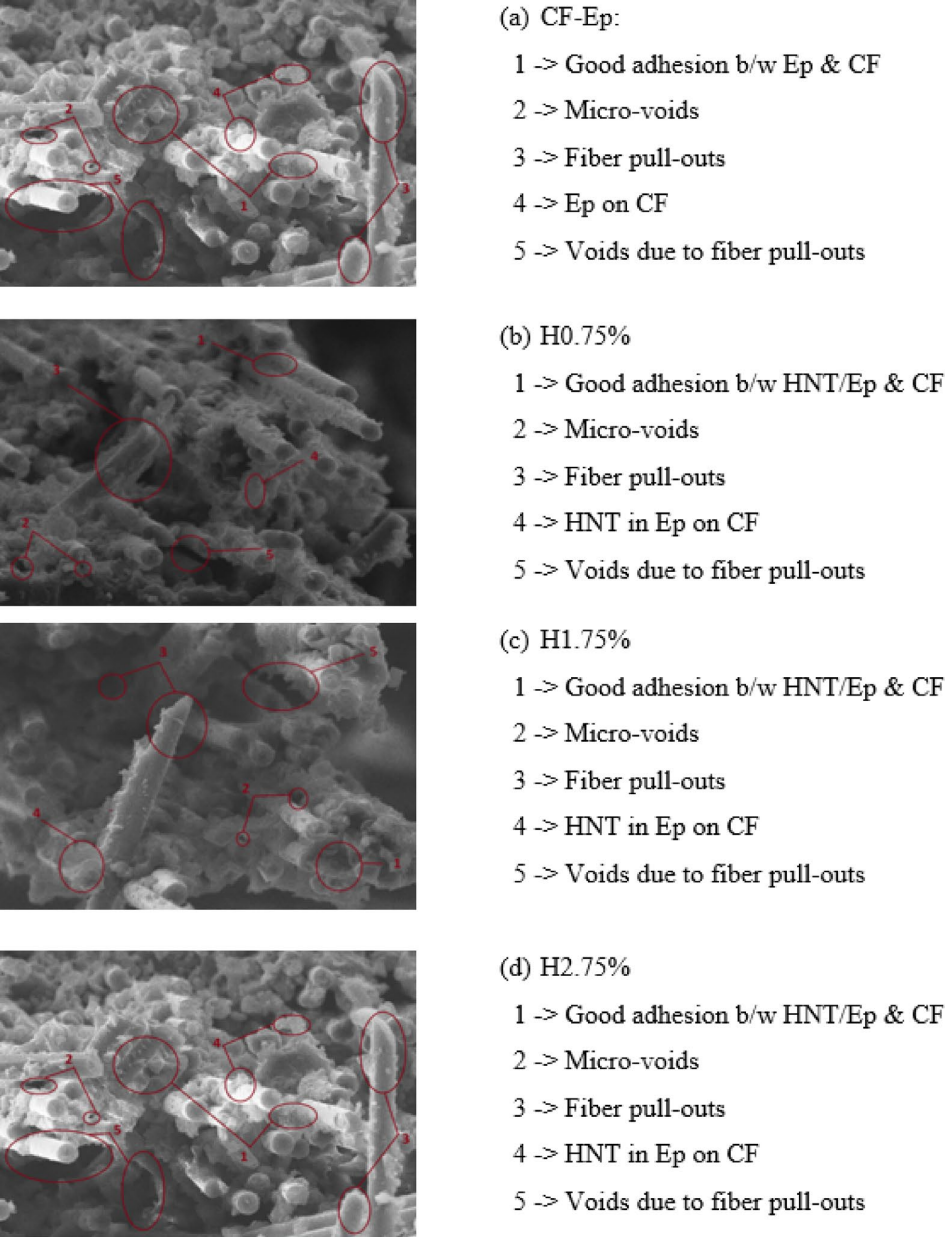
SEM micrographs of tensile-fractured surface of composites.

The SEM micrographs of tensile-tested composites (Fig. 8) revealed that the Ep matrix was predominantly covered with CFs. However, some evidence of micro-voids and fiber pull-outs was observed in all cases, including unfilled, H0.75%, H1.75%, and H2.75% CF-Ep composites^21,38,39^. Fractographic analysis of tensile-tested composites revealed primary failure mechanisms, including fiber pull-out, fiber breakage, and interfacial debonding^43,44^. The fractured surfaces showed evidence of adhesion between fiber and matrix, which may be mechanical or chemical in nature. Similar observations were reported by Ravichandran et al.^23^, where a coherent bond between Ep and HNT interface layers was observed, with minimal surface cavities. These findings suggest that interfacial bonding plays a crucial role in determining the tensile behavior of composites. The SEM analysis revealed a uniform distribution of HNTs in the CF-Ep composites up to a certain content, beyond which weak interfacial interactions and brittle cracking occurred. In this study, the smooth surface of the Ep-HNT-rich areas suggested strong packaging and absence of crack propagation. The fractured surface cross-sections (Fig. 8) showed homogeneous filler dispersion, fewer voids, and pull-outs, contributing to improved composite performance. The bright whitish matrix system indicated a strong cross-link among CFs, Ep matrix and HNTs. Compared to other filled and unfilled composites, less micro-voids and fiber pull-outs was observed in H0.75% composites contributing to improved composite performance.

Referring fractographic images depicted in Fig. 8, the addition of well-dispersed HNTs, achieved through the ultrasonication method, enhances interfacial bonding between the carbon fibers and epoxy matrix, effectively resisting interfacial debonding under stress. This stronger bonding facilitates efficient stress transfer, thereby reducing fiber pull-out and increasing fiber breakage during fracture. Moreover, the tubular structure of HNTs contributes to crack deflection and energy absorption, resulting in improved fracture toughness and more cohesive failure behavior.

The homogeneous dispersion and strong interfacial bonding between the HNTs and the epoxy matrix are responsible for the improved mechanical performance seen at 0.75 wt.% HNT. The presence of increasing HNT content was confirmed by EDAX spectra (Fig. 5b–d), but only the 0.75 wt.% composite showed effective distribution with no visible agglomeration. Reduced micro-voids and little fiber pull-out were revealed by SEM examination (Fig. 8b), suggesting better matrix integrity and stress transfer. Better load distribution and crack resistance are made possible by this uniform dispersion, which gives the H0.75% composite its highest tensile strength (820 MPa) and modulus (27 GPa). On the other hand, particle agglomeration was seen at larger HNT loadings (1.75% and 2.75%) (Fig. 8c, d), which resulted in localized stress concentrations, weakened interfacial bonding, and reduced mechanical characteristics. Therefore, the ideal concentration for optimizing composite performance was found to be 0.75 wt.% HNT.

### Differential scanning calorimetry

**Fig. 9:**
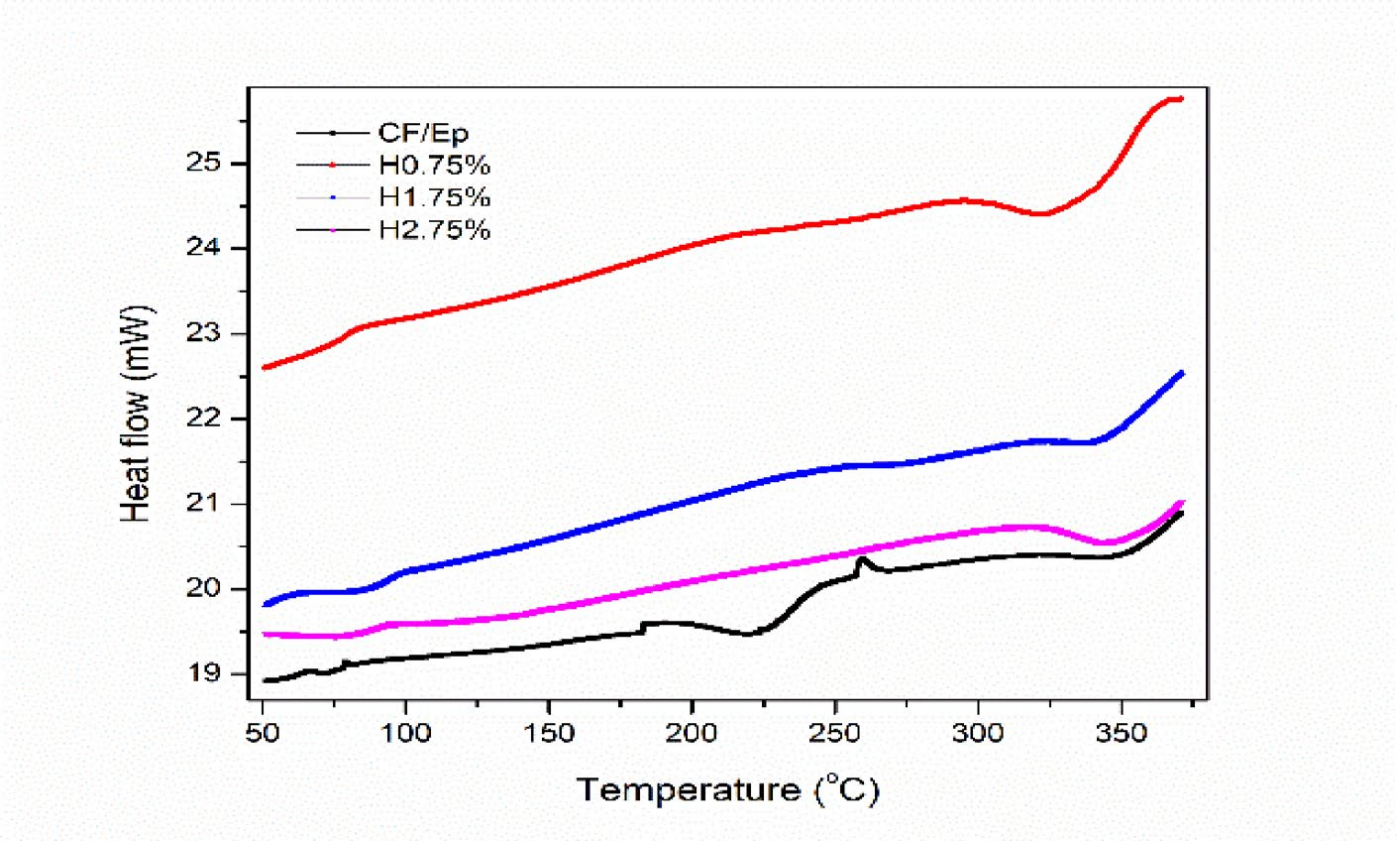
DSC thermograms of unfilled and HNT-filled composites.

Figure 9 presents the DSC curves of the composites. No significant exothermic transitions were observed in the H-CF-Ep samples, apart from minor peaks at 60 °C, 185 °C, and 270 °C. The main endothermic peaks, associated with the curing reaction, appeared around 342 °C, 315 °C, 335 °C, and 340 °C for unfilled, H0.75%, H1.75%, and H2.75% composites, respectively, indicating completion of curing.

**Fig. 10:**
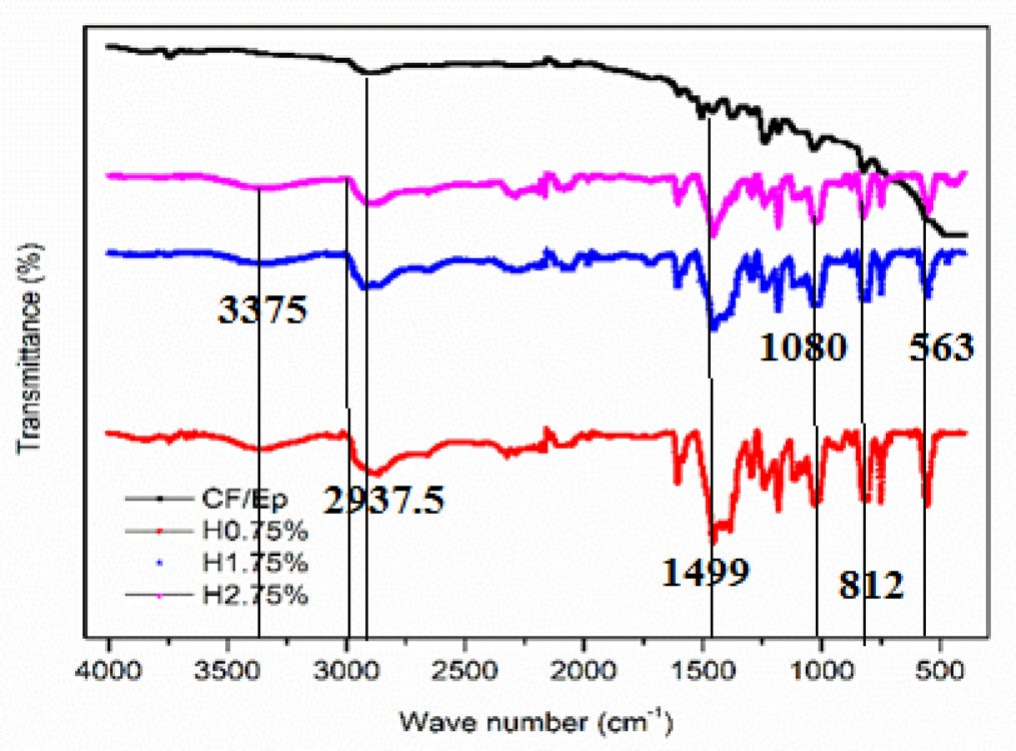
FTIR spectra of unfilled and HNT-filled composites.

These findings were further supported by FTIR analysis (Fig. 10). For clarity, the DSC curves were laterally shifted. Among all, the H0.75% composite showed the lowest endothermic peak and an almost linear curve, suggesting the shortest curing time and complete cure, as evidenced by the absence of a distinct exothermic peak. This enhanced curing likely contributes to its superior mechanical, dynamic-mechanical, and thermal stability. The degree of curing followed the trend: H0.75% > H1.75% > H2.75% > CF-Ep composites, which were consistent with FTIR, DMA, and TGA characterizations results.

### Fourier transform infrared spectroscopy

Figure 10 shows the FTIR spectra of both unfilled and HNT-filled composites. FTIR analysis was conducted to assess the addition and dispersion of HNT within the matrix. The characteristic peaks associated with the functional groups of the epoxy resin appear around 3380 cm^–1^, corresponding to hydroxyl and amine groups. These peaks are attributed to the O–H stretching vibrations, which become more prominent due to the formation of hydroxyl groups during the curing process^45,46^. Hydroxyl Groups (O–H) are the alcohols and phenols that exhibit a characteristic broad band in the 3200–3600 cm^–1^ range due to the O–H stretching vibration. The exact position and width of the band depend on the extent of hydrogen bonding between the hydroxyl groups. Amine Groups (N–H) are the primary amines (containing two N–H bonds) and amides (containing one N–H bond) that also show N–H stretching vibrations in the 3100–3500 cm^–1^ range. The peak at 3380 cm^–1^ could be attributed to the stretching of these bonds.

As previously established, the H0.75% composite demonstrated the best mechanical performance. In the HNT/epoxy spectra, a peak at 1117 cm^–1^ is linked to Si–OH groups (Silanol Groups (Si–OH) are hydroxyl groups that are attached to silicon atoms on the surface of silica materials. The presence of these groups indicates the surface is not fully condensed and may be involved in hydrogen bonding), while the peak at 908 cm^–1^ corresponds to Al–OH bending vibrations (Al–OH Bending are the Al–OH bond, like other bonds, can vibrate through stretching and bending. The bending vibration of the Al–OH group is commonly observed around 908 cm^–1^), both characteristic of HNT, aligning with earlier findings^47^. Additionally, the absorption band at 1031 cm^–1^ is attributed to Si–O stretching vibrations in HNT^48^. Si–O Stretching are the Si–O bond, strong covalent bond, and its stretching vibration (an elongation and contraction of the bond) appears in the infrared spectrum as a strong absorption band. Peaks at 1662 cm^–1^ and 565 cm^–1^ represent hydrogen bonding and Al–O–Si bending, respectively^49^. The presence of 1662 cm^–1^ peak confirms the successful incorporation of the polymer matrix and the formation of HNT-polymer interactions, potentially through hydrogen bonding. In aluminosilicates, silicon and aluminium atoms are typically tetrahedral coordinated, meaning they are surrounded by four oxygen atoms. These tetrahedral share corners, creates network structure. The Al–O–Si bending vibration refers to the deformation of the angle between these tetrahedral. The presence of these Al, Si, and OH-related peaks provides evidence for the successful addition and even dispersion of HNTs within the epoxy matrix. FTIR analysis revealed similar vibration bands and peaks for all composites, with variations in peak intensities. The HNT-filled composites exhibited additional peaks due to the presence of HNT. Notably, H0.75% composites showed more pronounced and sharper absorption peaks, indicating better mechanical behavior, mixing, curing, and dispersion of nano-HNT in the epoxy system. The high-intensity peaks in H0.75% composites were attributed to efficient cross-link density formation. Conversely, unfilled composites demonstrated decreased peak intensities, resulting from poor cross-linking density and unreactive diluents.

### Dynamic mechanical analysis

#### Storage modulus

**Fig. 11:**
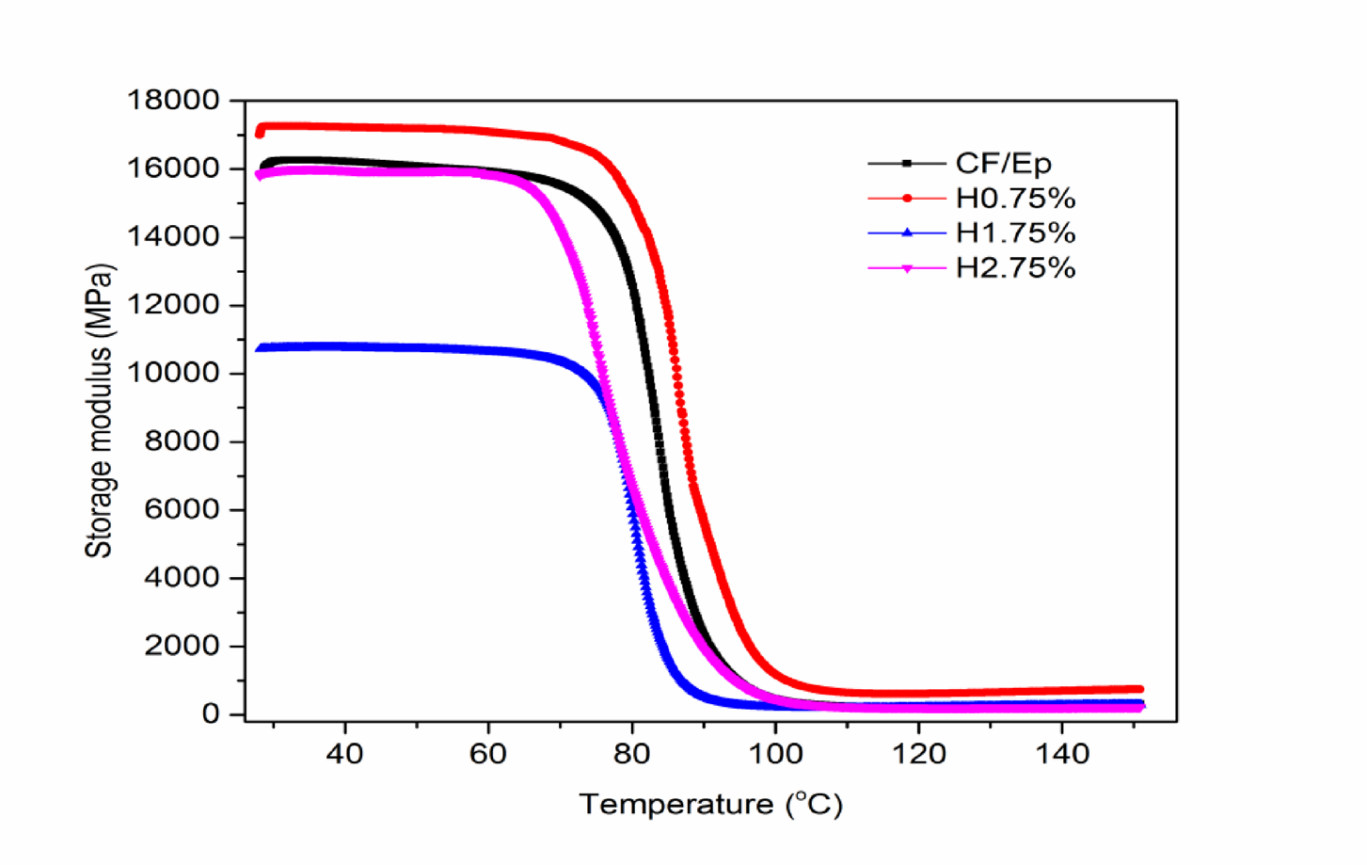
Storage modulus of unfilled and HNT-filled composites.

The storage modulus of composites with and without HNT filler, as shown in Fig. 11, exhibits a dependence on temperature and filler content. The unfilled CF-Ep composite has an initial storage modulus of approximately 16,000 MPa, which decreases with increasing temperature and drops sharply at around 90 °C, marking the onset of the glass transition region. For the unfilled composite, the storage modulus remains constant from 27 to 75 °C, followed by a decline until 90 °C, and then remains constant until 273 °C. Similarly, the HNT-filled composites (H0.75%, H1.75%, and H2.75%) exhibit initial constant storage modulus ranges of (27–78) °C, (27–73) °C, and (27–66) °C, respectively, followed by a decline and a rubbery regime. The composite with 0.75% HNT exhibits the highest storage modulus across all temperatures, suggesting effective HNT dispersion and strong bonding with the Ep matrix. This enhancement in stiffness likely results from improved load transfer and resistance to thermal stress. In contrast, the composites with 1.75% and 2.75% HNT show reduced storage modulus, potentially due to less uniform dispersion, microstructural defects, clumping, and poor interaction with the matrix. The optimal HNT content of 0.75% provides the best mechanical performance improvement, consistent with previous tensile and FTIR results. This is attributed to better dispersion and interaction with the matrix. Furthermore, the storage modulus and tensile strength are higher for the H0.75% composite, indicating a correlation between temperature and strength, as also reported by Bosze et al.^9,50^.

#### Loss modulus

**Fig. 12:**
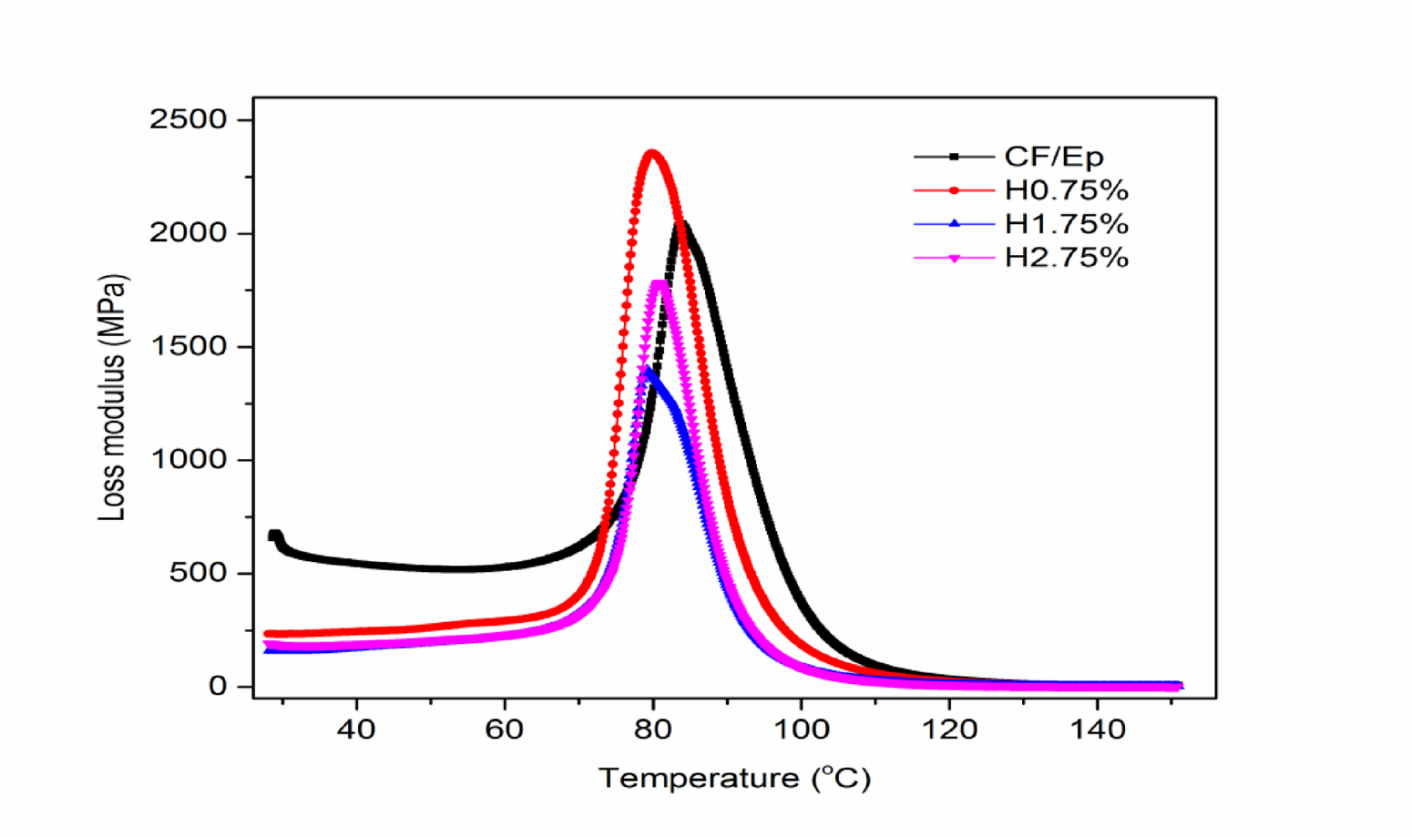
Loss modulus of unfilled and HNT-filled composites.

Figure 12 shows the loss modulus (LM) curves of composites with varying HNT content. The H0.75% composite exhibited the highest LM values. The LM values remained stable at lower temperatures, followed by a sharp increase around 60–70 °C, reaching a peak, and then decreasing sharply. The peak LM temperatures were 85 °C for the unfilled composite, 81 °C for H0.75%, 77 °C for H1.75%, and 80 °C for H2.75%. The LM curves followed the trend H0.75% > unfilled > H2.75% > H1.75%, with H0.75% composites showing the best LM values^9^. These results are consistent with Muralidhara et al., who reported that unfilled composites can outperform composites with high filler content (2.75 wt.% GnP)^37^.

#### Damping factor

**Fig. 13:**
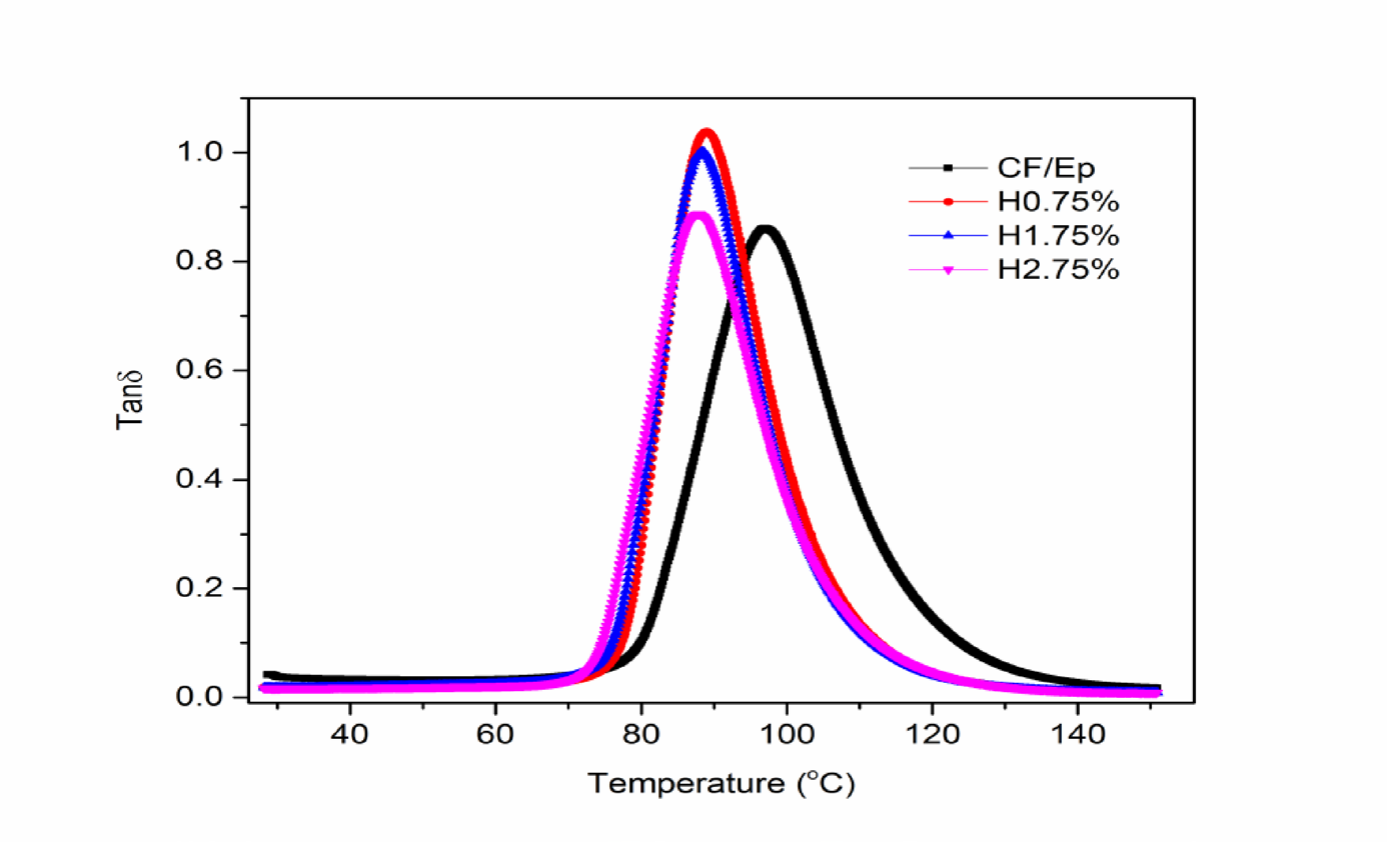
Damping factor of unfilled and HNT-filled composites.

Figure 13 shows the damping coefficient (DC) curves of the composites. The DC curves exhibit a rapid increase around 70 °C, reaching a peak, followed by a steep decline, and then stabilizing at approximately 140 °C. The H0.75% composite showed higher DC values compared to the unfilled composite, indicating the impact of HNT addition on the Ep matrix. Similar findings were reported by Hariharasudhan et al. in their study on nano clay-filled CF-Ep composites^9^. The glass transition temperature (T_g_) was calculated using the tanδ peak values, a widely accepted method. Previous studies have shown that T_g_ can be enhanced by adding fillers, but only up to a certain limit.

Excessive filler content can lead to a decline in T_g_ due to reduced cross-linking density and increased free volume in the matrix system^51–55^. In this study, the T_g_ values for the unfilled and HNT-filled CF-Ep composites were 97.07 °C, 89 °C, 88.43 °C, and 87.8 °C, respectively. The unfilled composite exhibited the highest T_g_, while the H2.75% composite showed the lowest operating temperature due to its reduced visco-elastic properties and operating life. These findings are consistent with previous research indicating that higher mechanical performance does not always translate to higher T_g_ values^7,37^. Moreover, in H0.75% HNT-filled composites, the tanδ peak is higher and slightly shifted toward a higher temperature, suggesting improved energy dissipation and a higher glass transition temperature (T_**g**_). This implies stronger filler–matrix contact, improved thermal stability, and improved damping behavior all of which improve mechanical performance.

### Thermogravimetric analysis

**Fig. 14:**
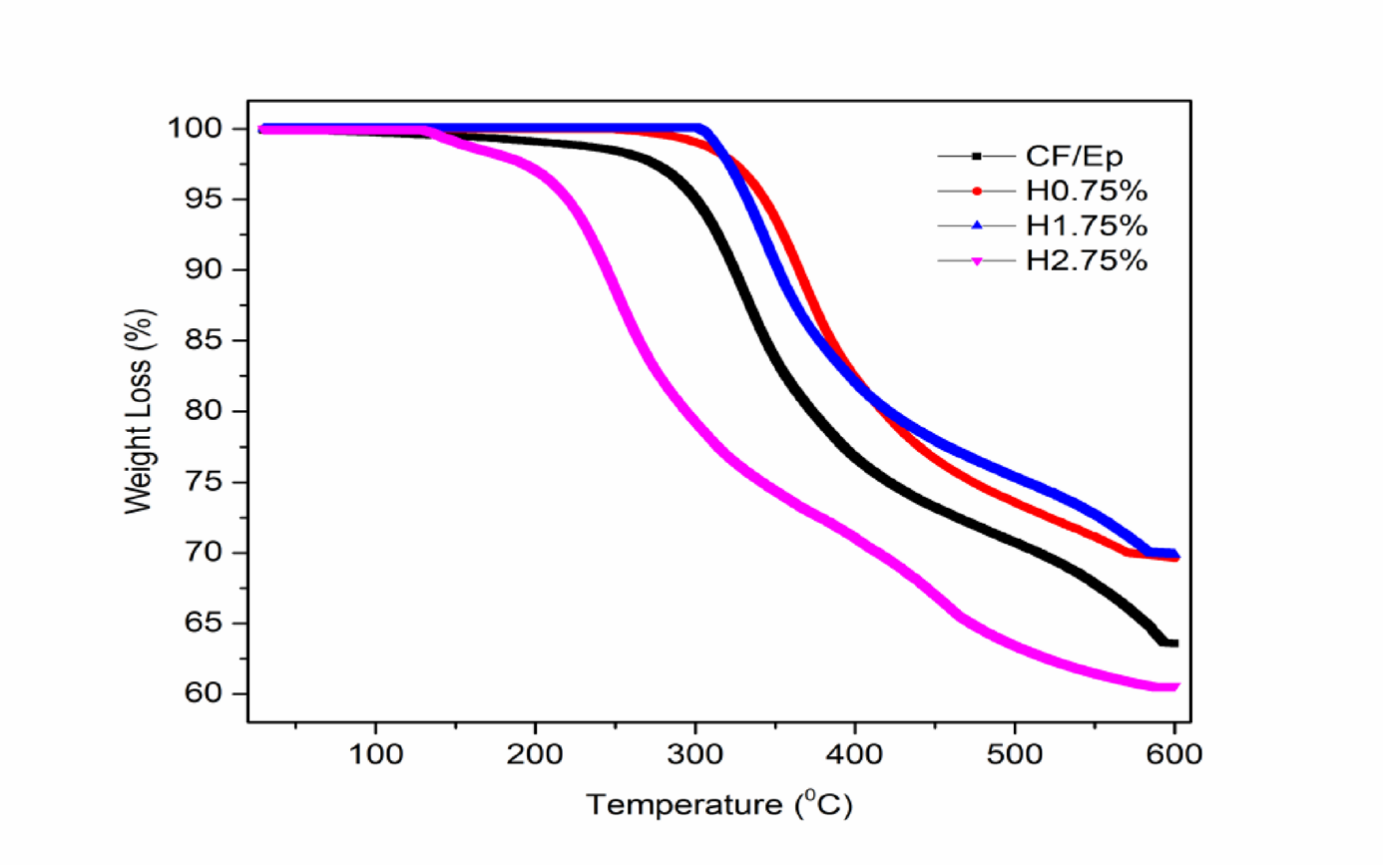
Wear loss of unfilled and HNT-filled composites.

The thermogravimetric analysis (TGA) curves of unfilled and HNT-filled CF-Ep composites are displayed in Fig. 14, which shows how the amount of halloysite nanotube (HNT) affects thermal stability. Because HNTs are ceramic-like and can function as thermal barriers, it was expected that their addition would improve the composites’ overall thermal resistance. The stability of the H0.75% and H1.75% composites was better, and the main degradation onset was postponed to around 320–325 °C as opposed to 150 °C in the unfilled system. By preventing the release of volatile degradation products and improving matrix–filler interactions, HNTs can prevent thermal decomposition, according to these findings^56^. With an onset degradation behavior resembling that of the unfilled CF-Ep composite, the H2.75% composite performed abnormally, deteriorating earlier than other HNT-filled systems and displaying the most severe and quick weight loss^57^. The dispersion and interfacial limitations that occur at higher HNT loadings provide a reasonable explanation for this apparent discrepancy with the widely held belief that increased filler content enhances thermal stability. The epoxy matrix becomes oversaturated with nanotubes at 2.75 wt.%, surpassing the dispersion capacity attained by ultrasonication. As a result, HNTs aggregate, creating voids and micro-clusters that jeopardize the matrix’s structural consistency. These agglomerates act as preferred sites for the initiation of degradation because they not only break up the continuous network of the polymer but also introduce thermal weak spots. Furthermore, a high HNT content can decrease the overall interfacial bonding between the epoxy and filler and impede effective curing. The protective effect of HNTs during thermal loading is compromised by their poor matrix–filler adhesion, which restricts their capacity to act as efficient barriers against heat and mass transfer. Because of structural flaws and poor interfacial integrity, the H2.75% composite shows lower thermal resistance even though it contains higher filler content. These findings align with earlier studies which emphasized that there is an optimal nanoparticle concentration beyond which thermal and mechanical performance deteriorates due to aggregation effects^1,58–60^. Similar results were found by Muralidhara et al. in graphene nanoplatelets-reinforced CF–Ep composites, where a high intrinsic heat resistance of the nanofillers was accompanied by decreased thermal stability due to excessive filler content^37^.

In a nutshell filler agglomeration, interfacial defects, and compromised matrix cohesion cause the H2.75% composite to exhibit lower thermal performance even though higher HNT loading is generally linked to improved thermal stability. For better thermal durability, it is crucial to optimize the filler content in order to achieve uniform dispersion and an efficient matrix–filler interaction.

## Wear behavior

### Effect of HNTs loading on the dry sand abrasion of CF-Ep composites

**Fig. 15:**
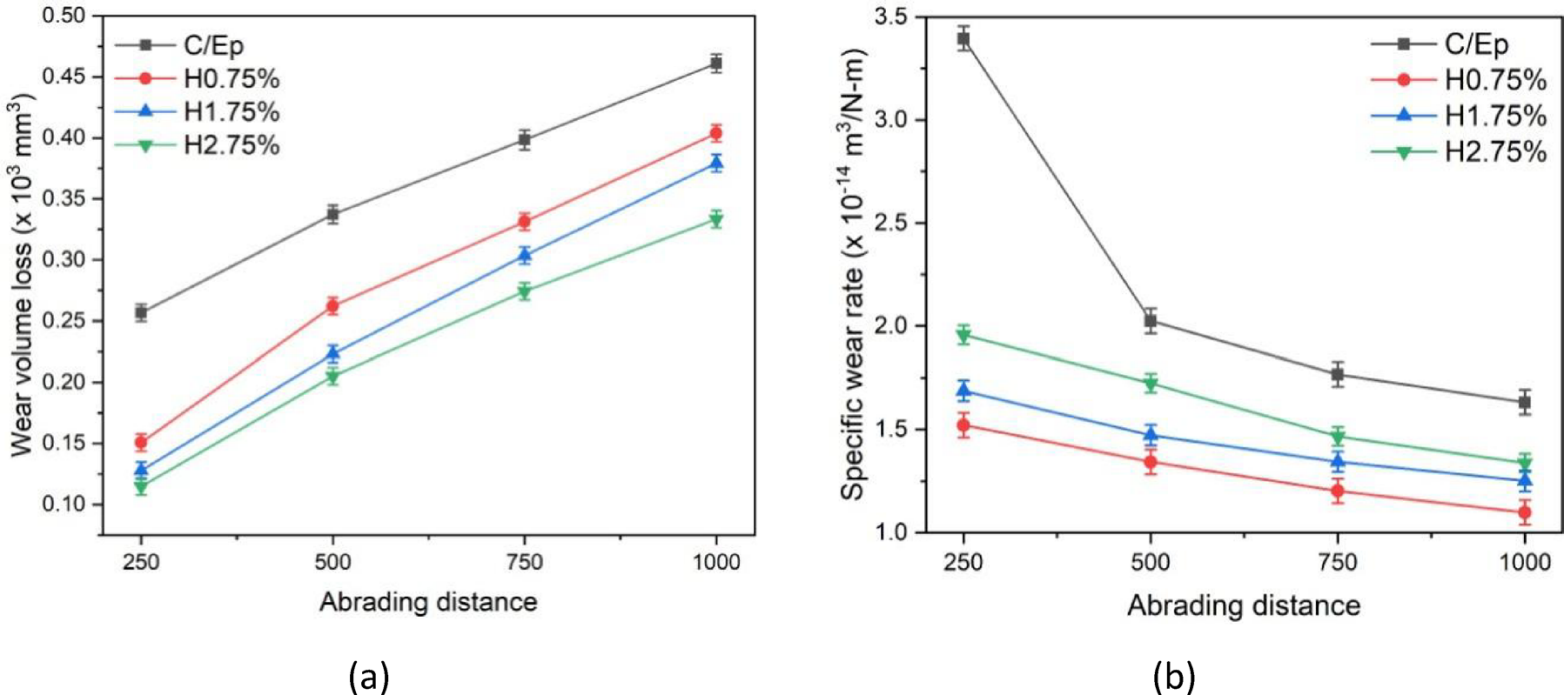
Variation in (**a**) wear loss and (**b**) SWR of unfilled and HNT-filled composites.

**Fig. 16:**
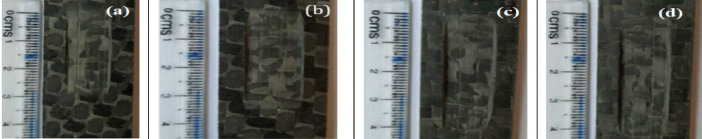
Photographs of H0.75% composites (after subjected to TBA for constant load 30 N and variable distances (**a**) 250 m, (**b**) 500 m, (**c**) 750 m and (**d**) 1000 m).

Figure 15 illustrates the variation in wear loss (WVL) and sp. wear rate (SWR) for both unfilled and HNT-filled composites. It is observed that the inclusion of HNT in CF-Ep composites significantly enhances wear resistance. As the distance increases, WVL rises while SWR decreases^61–63^. In the WVL curves for both unfilled and HNT-filled composites, the trend observed is CF-Ep > H2.75% > H1.75% > H0.75%. The WVL values for CF-Ep, H2.75%, H1.75%, and H0.75% composites increased from 0.255 to 0.46 mm^3^ × 10^3^, 0.145 to 0.395 mm^3^ × 10^3^, 0.126 to 0.375 mm^3^ × 10^3^, and 0.11 to 0.325 mm^3^ × 10^3^, respectively. Similarly, in the SWR curves, the trend remains CF-Ep > H2.75% > H1.75% > H0.75%. The SWR values for CF-Ep, H2.75%, H1.75%, and H0.75% composites decreased from 3.4 to 1.7 m^3^/N-m × 10^–14^, 1.95 to 1.3 m^3^/N-m × 10^–14^, 1.75 to 1.35 m^3^/N-m × 10^–14^, and 1.52 to 1.1 m^3^/N-m × 10^–14^, respectively. Suresha et al. demonstrated that Ep composites with low wt.% of BC filler exhibit superior three-body abrasive wear resistance compared to those with higher filler content^63^. Similarly, this study concludes that H0.75% composites outperform others in both WVL and SWR results. Figure 16 presents normal photographs of H0.75% composites subjected to varying distances ranging from 250 to 1000 m in 250 m increments^41^.

#### Effect of HNT loading on the friction and wear of CF-Ep composites

**Fig. 17:**
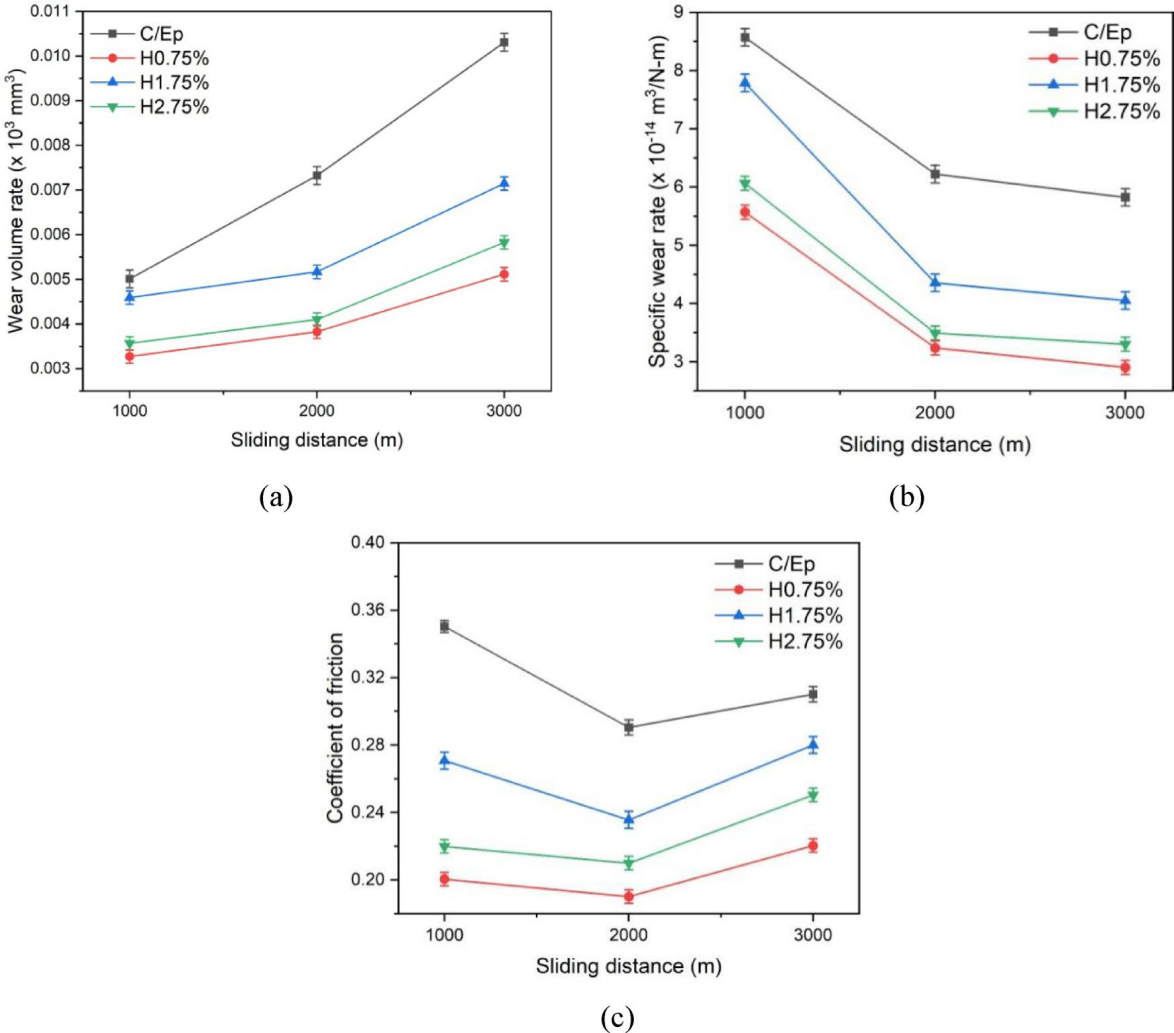
Comparative analysis of (**a**) wear volume loss (WVL), (**b**) specific wear rate (SWR), and (**c**) coefficient of friction (COF) for unfilled and HNT-reinforced composites under dry sliding wear conditions.

Figure 17 illustrates the relationship between wear rate and sliding distance in a dry sliding wear test. The results indicate that incorporating HNT into CF-Ep composites significantly enhances their wear resistance. As the sliding distance increases, the WVL rises while the SWR declines. As seen in the figure, H0.75% composites achieved superior WVL and SWR outcomes compared to others. Previous studies by Suresha et al. and Veena et al. have demonstrated that adding hard particles like BC_3_, TiO_2_, Al_2_O_3_, ZrO, SiO_2_, and SiC to PMC improves wear resistance^63–65^.

Similarly, this study found that adding HNT significantly improved wear resistance in nano-HNT filled-CF-Ep composites compared to unfilled ones, with WVL and SWR curves (in terms of worn material removal) following the trend: CF-Ep > H1.75% > H2.75% > H0.75% composites. The WVL values for CF-Ep, H0.75%, H1.75%, and H2.75% composites increased from (0.0048–0.012 mm^3^ × 10^3^), (0.00325–0.0052 mm^3^ × 10^3^), (0.0046–0.007 mm^3^ × 10^3^), and (0.0034 to 0.006 mm^3^ × 10^3^) respectively, while the SWR values for unfilled, H0.75%, H1.75%, and H2.75% CF-Ep composites decreased from (8.6–6.2 m^3^/N-m × 10^–14^), (5.6–2.95 m^3^/N-m × 10^–14^), (7.75–4.2 m^3^/N-m × 10^–14^), and (6.05–3.3 m^3^/N-m × 10^–14^) respectively.

**Fig. 18:**
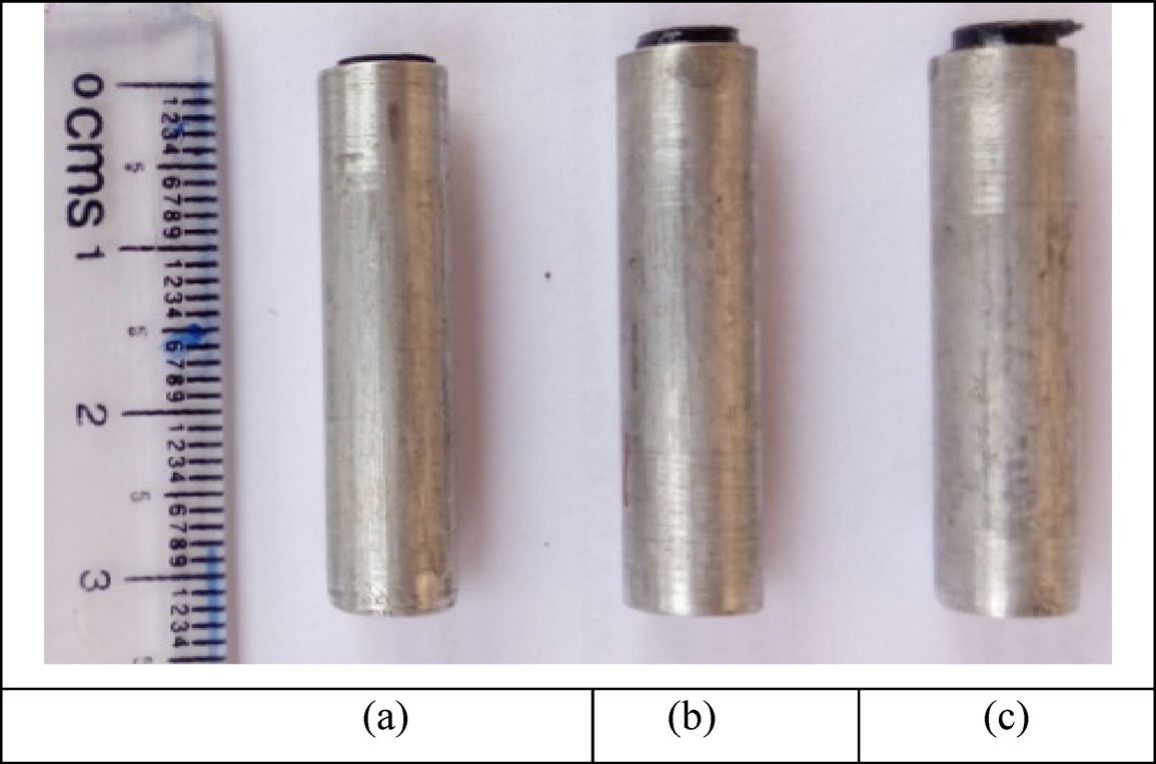
Photos of H0.75 composites pin specimen (**a**) 3000 m, (**b**) 2000 m, and (**c**) 1000 m.

Figure 18 shows the worn surfaces of H0.75% composite samples after pin-on-disc (POD) tests at varying sliding distances of 1000 m, 2000 m, and 3000 m. The images indicate that material removal increases with sliding distance, consistent with previous studies. The 1000 m distance tested samples showed less fragmentation and material removal, resulting in lower wear. The coefficient of friction (COF) curves shows an initial increase up to 1000 m, followed by a decrease at 2000 m, and then an increase at 3000 m. The COF trend was CF-Ep > H1.75% > H2.75% > H0.75% composites. Specifically, the COF values of CF-Ep, H0.75%, H1.75%, and H2.75% composites decreased from 1000 to 2000 m and then increased from 2000 to 3000 m, with values ranging from 0.35 to 0.292 to 0.31, 0.20 to 0.19 to 0.225, 0.27 to 0.237 to 0.282, and 0.22 to 0.212 to 0.25, respectively. These findings are consistent with Mishra et al.^66^, who reported that COF increases up to a certain value and then decreases with further increase in normal load or sliding velocity. The sliding distance significantly influences the COF of the composites.

Halloysite nanotubes’ distinct nanoscale shape and surface chemistry are responsible for the enhanced tribological performance shown with HNT inclusion, especially at 0.75 wt.%. Due to their hollow tubular structure and high aspect ratio (Table 1: 1–15 μm in length and 10–30 nm in diameter), HNTs improve mechanical interlocking within the epoxy matrix and aid in efficient stress transfer during sliding. HNTs have a chemically heterogeneous surface that encourages good interfacial adhesion with the epoxy matrix because the inner lumen includes aluminol (Al–OH) groups and the outer surface is rich in siloxane (Si–O–Si) groups. Under tribological loads, this robust interfacial bonding reduces fiber-matrix debonding and micro-crack propagation.

Furthermore, by reducing direct contact between mating surfaces, well-dispersed HNTs function as nanoscale bearing elements, lowering the coefficient of friction and specific wear rate. HNTs are evenly distributed at the ideal loading of 0.75 wt.%, which makes it easier for a stable and protective transfer layer to form during sliding. Surface damage and material removal are reduced using this tribo-film. Partial agglomeration, on the other hand, may happen at higher loadings (e.g., Fig. 17: 1.75% and 2.75%), leading to stress concentrations and decreased wear resistance. Therefore, improving the composite’s wear resistance under dry sliding conditions requires a synergy between HNT size, dispersion quality, and interfacial interaction.

#### ANOVA on TBA wear tests

**Fig. 19:**
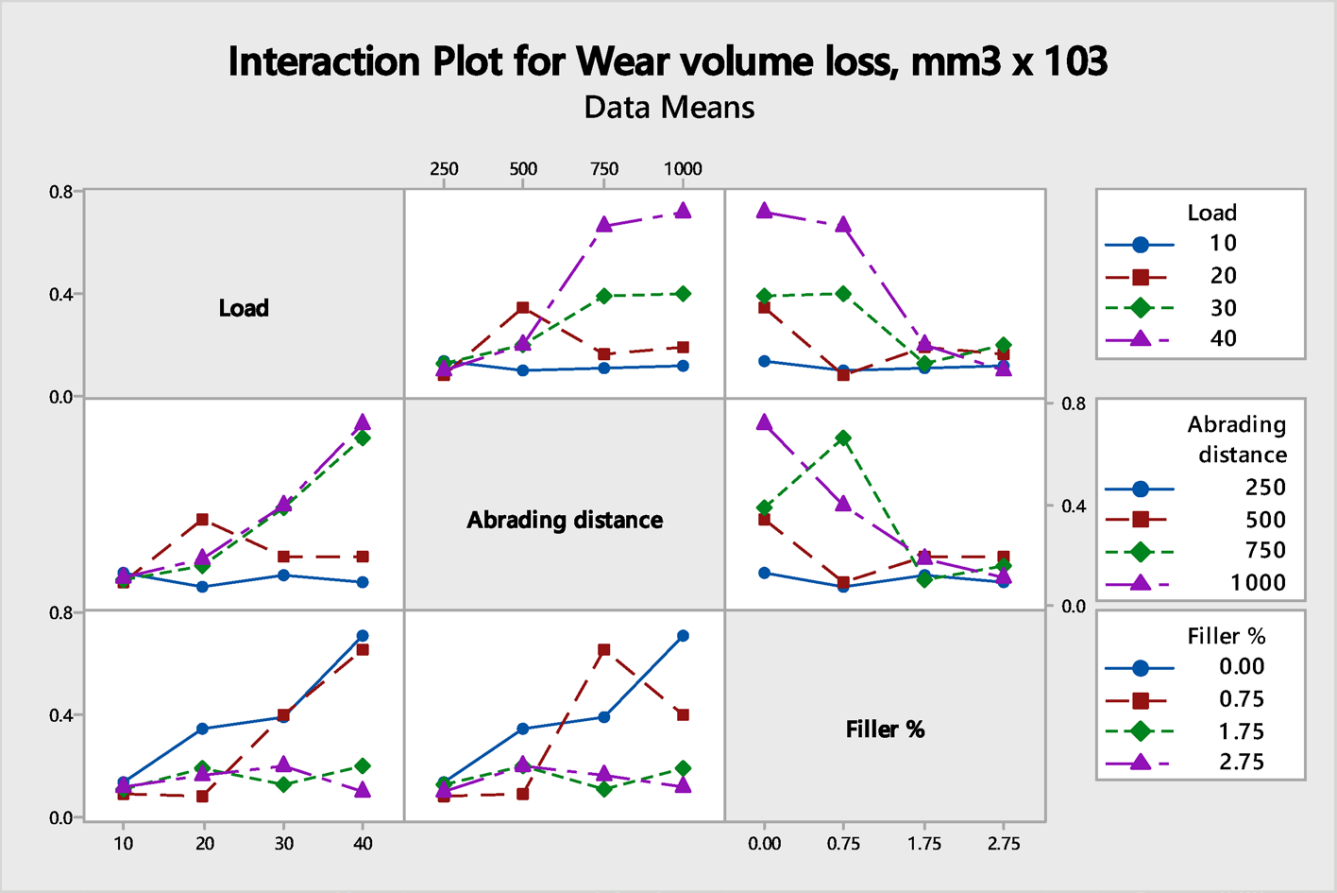
Interaction plot for WVL of 212-HNT composites.

**Fig. 20:**
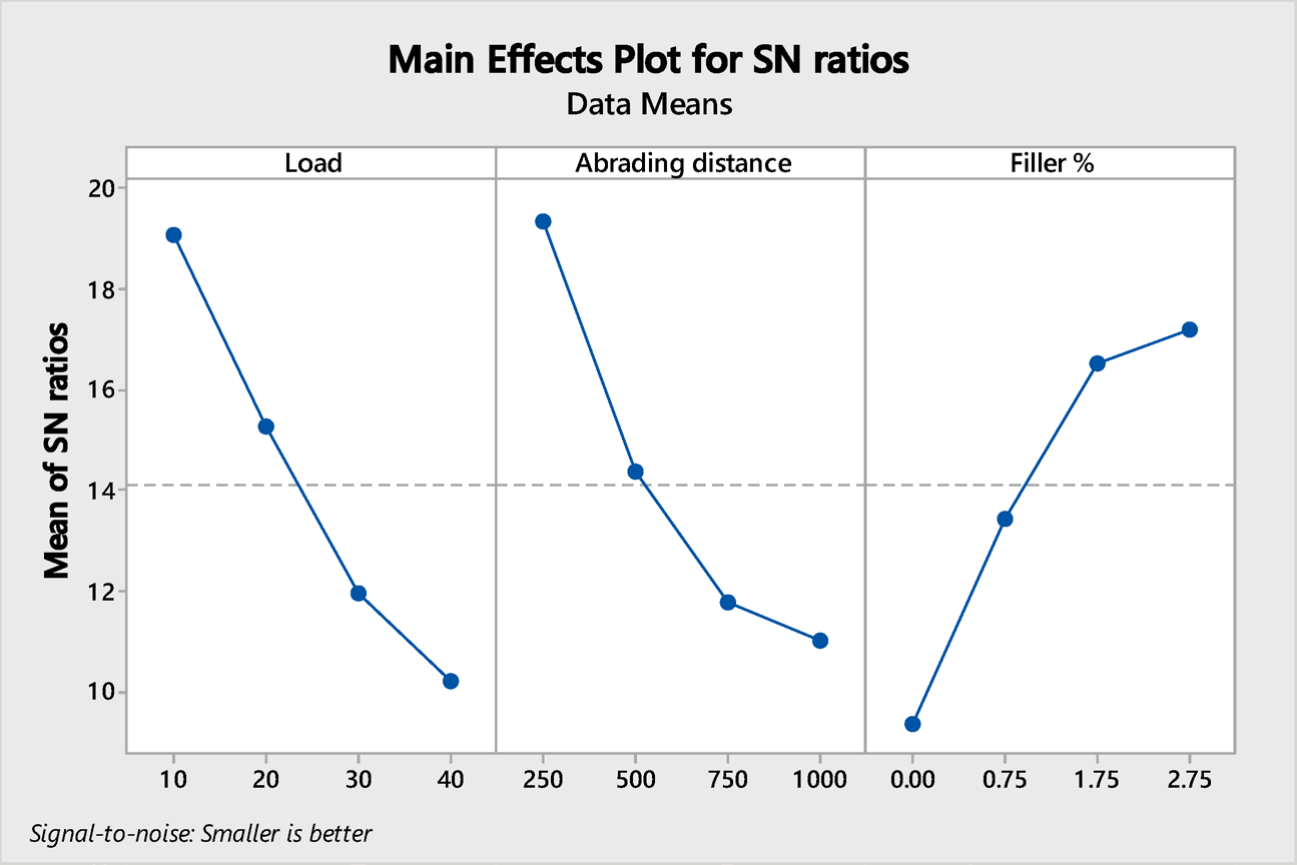
S/N ratio main effects plot for WVL of 212-HNT composites.

Sixteen wear tests were performed by three-body abrasive wear testing machine following the DOE to study and analyse the abrasive wear of micro-sized 212-HNT composites by varying the three factors such as load, abrading distance and filler wt.%. Based on the results, a mathematical model was developed using MINITAB 17 software. For the 212-HNT composites, the developed model yielded an adjusted R^2^ value of 89.75%, indicating a strong correlation between the experimental and predicted results. Tables 3 and 4 shows the significance of control factors on abrasive wear that was analyzed using S/N ratio response. The interaction and S/N ratio main effect plots for the WVL of the process parameters on 212-HNT composites are as shown in Figs. 19 and 20.

**Table 4:** Standard orthogonal L_16_ array with output results for WVL of 212-HNT composites.

Expt. No	Load (N)	Abrading distance (m)	Filler wt.%	S/N Ratio	Expt. WVL	Theor. WVL	Error (%)
1	10	250	0.00	17.3548	0.13560	0.114	15.93
2	10	500	0.75	20.5912	0.09342	0.1018	8.97
3	10	750	1.75	19.7012	0.10350	0.0856	17.29
4	10	1000	2.75	18.7258	0.11580	0.06635	> 20
5	20	250	0.75	21.6520	0.08268	0.1708	> 20
6	20	500	0.00	9.1535	0.34860	0.234	> 20
7	20	750	2.75	15.7456	0.16320	0.14335	12.16
8	20	1000	1.75	14.5030	0.18830	0.217325	15.41
9	30	250	1.75	17.9034	0.12730	0.151575	19.06
10	30	500	2.75	13.8887	0.20210	0.14035	> 20
11	30	750	0.00	8.0615	0.39530	0.434	9.79
12	30	1000	0.75	7.9328	0.40120	0.4433	10.49
13	40	250	2.75	20.4455	0.09500	0.05735	> 20
14	40	500	1.75	13.8758	0.20240	0.283325	> 20
15	40	750	**0.75**	3.5305	0.6660	0.623175	6.43
16	40	1000	0.00	2.8678	0.71880	0.714	0.66

The importance of each factor was analyzed from the S/N ratio main effect plot’s inclination. In the interaction plots, a combined factor for which the inclined line has the highest elevation will have the most significant. In main effect plots, the individual factor for which the inclined line has the best elevation will be most effective. Table 5 indicates the (load + abrading distance) and (load + filler) has the most significant effect on the WVL of 212-HNT composites. The pooled error associate in the ANOVA is 10.24%. The S/N ratio main effect plots indicate that individually abrading distance, load and filler also have some effect in those composites.

**Table 5:** ANOVA results for S/N ratio of 212-HNT composites.

Source	DF	Adj SS	Adj MS	F	*P*	Pr %
Load (N)	1	0.04010	0.04010	1.6687	< 0.05	6.61
Abrading distance (m)	1	0.04640	0.04640	1.9393	< 0.05	7.66
Filler %	1	0.02110	0.02110	0.9020	> 0.05	3.48
(Load + Distance)	1	0.25350	0.25350	10.5534	< 0.05	41.80
(Load + Filler)	1	0.18290	0.18290	7.6219	< 0.05	30.15
(Distance + Filler)	1	0.00030	0.00030	0.0015	> 0.05	0.05
Error	9	0.06210	0.00621			10.24
Total	15	0.606483				100

The multiple linear regression equation for 212-HNT composites (Eq. 3) is developed. The linear regression equation developed for WVL of above composites is as given below:

3 $${\text{Wear}}\,{\text{volume}}\,{\text{loss}},{\text{mm}}^{{3}} \times {1}0^{{3}} = \left( {0.0{74} + 0.00{\text{48L}}{-}0.000{\text{192D}} + 0.0{\text{349F}} + 0.0000{\text{16L}}*{\text{D}}{-}0.00{\text{375L}}*{\text{F}}} \right)$$

**Fig. 21:**
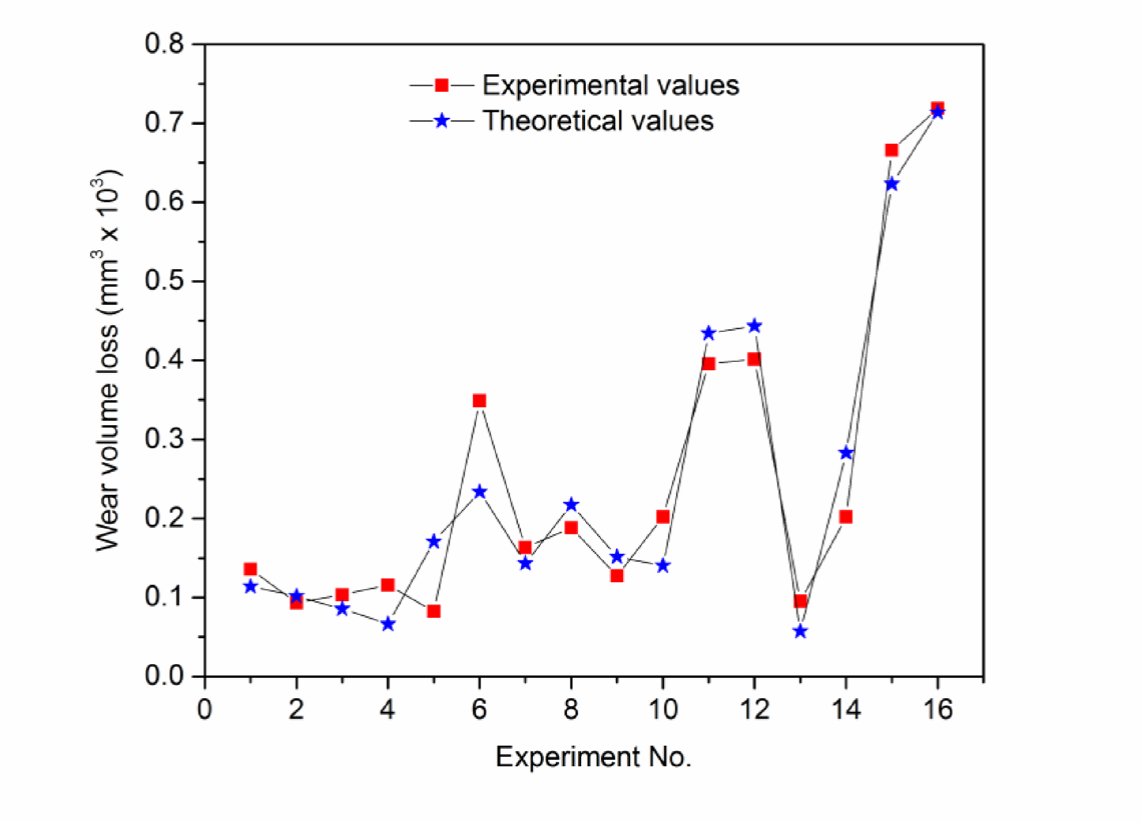
Experimental and theoretical values of WVL of 212-HNTcomposites.

The theoretical WVL was calculated by using the Eq. (3) and the values are plotted as shown in the Fig. 21, where experimental and theoretical values are seen closer to each other.

#### Confirmation experiment

A confirmation test using selected combinations of the factors and levels provide validation and accuracy of this analysis. The selected combination of factors/levels for confirmation experiment was chosen other than the combination selected for the already performed L_16_ orthogonal array of the three-body tests.

At the final stage, the performance characteristics were confirmed using the level of design factors (L_30_ D_1000_ F_1.75_) and (L_30_ D_1000_ F_2.75_) in 212-HNT composites. The theoretical regression WVL was calculated using Eq. (3). The experimental values of WVL for 212-HNT are 0.2547 and 0.1291 respectively, whereas the theoretical regression values of WVL for 212-HNT composites are calculated to be 0.2732 and 0.1182 respectively as shown in Table 6. The predicted WVL are very close to the actual values in 212-HNT composites. When the comparison was made between the experimental and theoretical WVL, by considering the selected combination of factors/levels, the difference between the experimental and theoretical results are very close to the satisfactory range of 5% error. From these confirmation results, it is shown that 212-HNT composites will give the better wear resistance in terms of WVL and also, this output response model can be efficiently utilized to analyse and predict the wear behaviour.

**Table 6:** Confirmation experiment.

Process parameters		Expt. WVL	Theor. regression WVL	Error (%)
HNT-212 composites	L_30_ D_1000_ F_1.75_	0.2547	0.2732	7.26
	L_30_ D_1000_ F_2.75_	0.1291	0.1182	8.53

#### Worn-out morphology

**Fig. 22:**
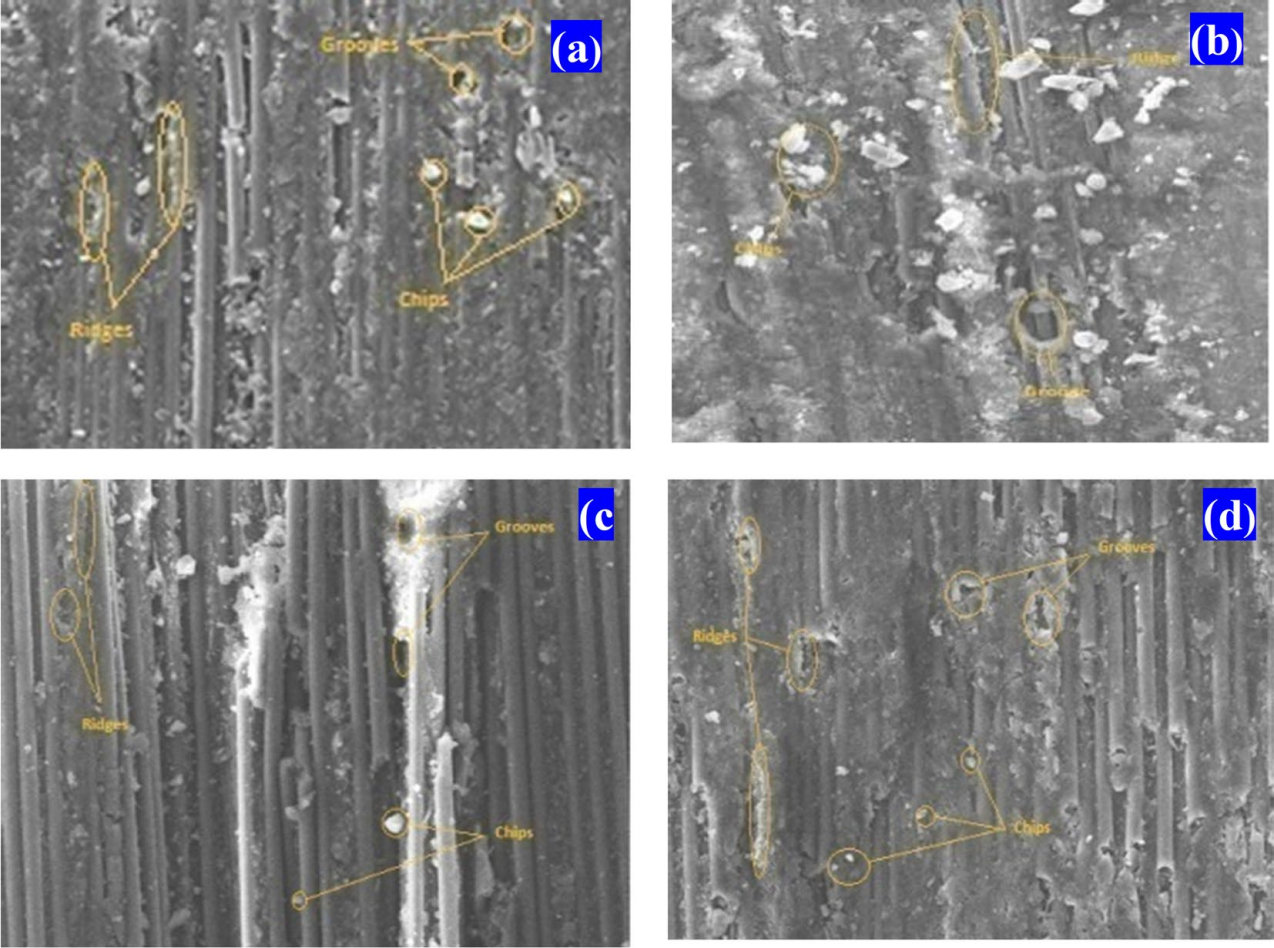
SEM worn-out morphology of composites under TBA test (30 N load and 1000 m distance) (**a**) CF-Ep, (**b**) H0.75%, (**c**) H1.75%, (**d**) H2.75%

Figure 22 presents SEM micrographs of unfilled, H0.75%, H1.75%, and H2.75% CF-Ep composites after tribological tests. The micrographs reveal key factors influencing abrasion, including micro-ploughing, micro-cutting, and fragmentation. The worn surfaces show evidence of micro-ploughing, characterized by grooves and ridges, as well as micro-cutting, indicated by micro-chips and debris. Fragmentation is also evident, with fractures, wear indentations, and micro-cracks observed in the micrographs. The larger abrading distance led to deterioration of the top matrix layers, weakening the fiber-matrix bonding, and resulting in matrix detachment. The presence of asperities facilitated layer removal, and the friction-induced removal of matrix, debris, and sand particles protected the fibers for a longer duration. Notably, the HNT nanoparticle-filled composites demonstrated significant effectiveness. The high wear resistance of H0.75% composites can be attributed to reduced micro-ploughing, cracking, and cutting of surface asperities, consistent with previous studies^61,66–68^.

**Fig. 23:**
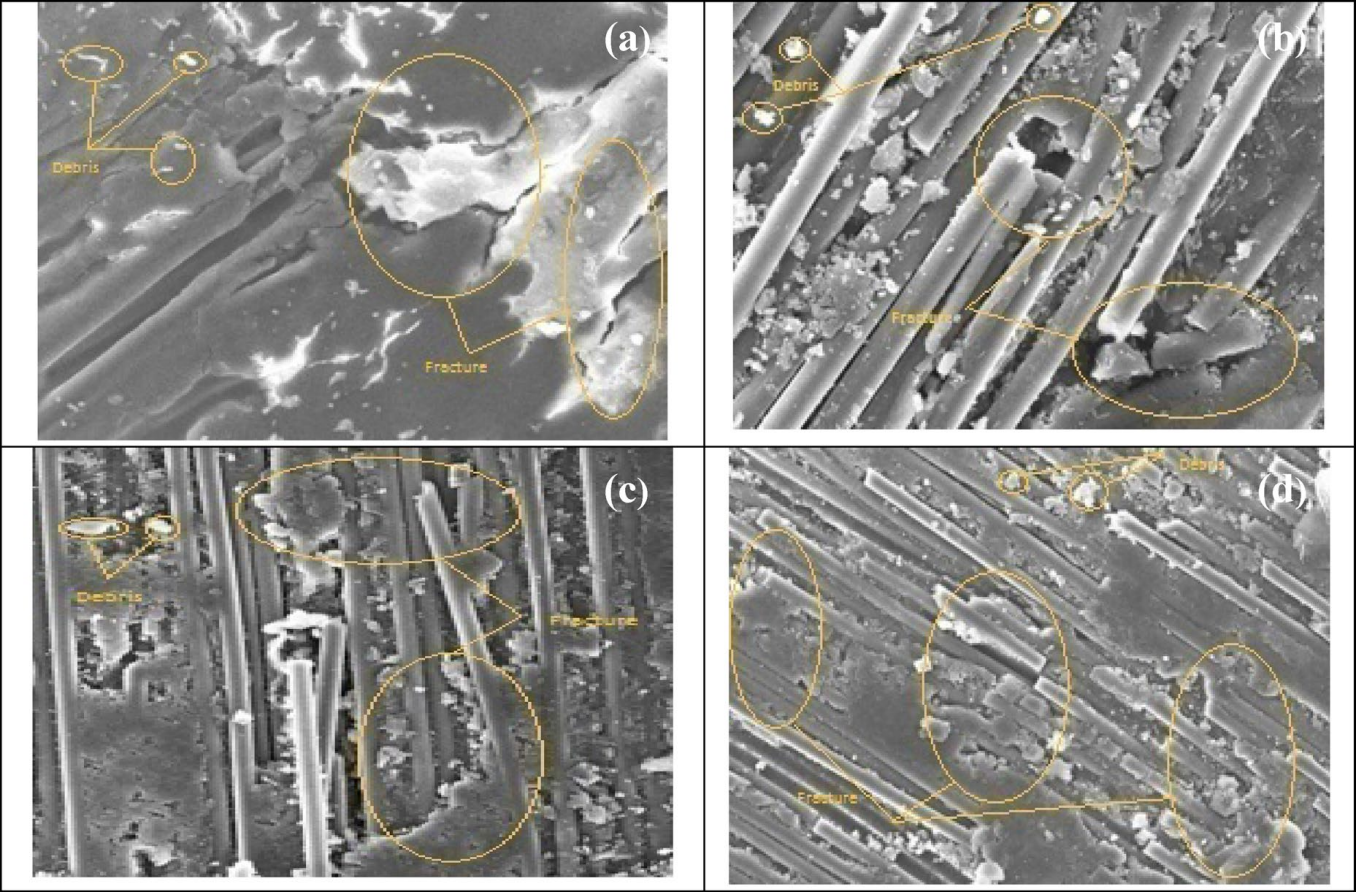
SEM worn-out morphology of composites under DSFW tests, 30 N load and 1000 m distance: (**a**) CF-Ep, (**b**) H0.75%, (**c**) H1.75%, and (**d**) H2.75%

Figure 23 shows SEM micrographs of worn surfaces of composites subjected to dry sliding wear. The wear behavior of unfilled composites exhibits significant material pull-out, whereas filled composites show improved wear resistance. The addition of fillers, such as SiO_2_ or HNTs, acts as secondary reinforcement, bearing considerable load and reducing wear rate. Uniformly dispersed fillers enhance wear resistance by forming a protective layer that prevents large-scale fragmentation of the epoxy matrix. In this study, SEM images reveal material removal through fragmentation, with solid-phase welding of surface particles resulting in material transfer due to electrostatic forces and continuous shearing. The adhesive wear images show that high filler content can lead to agglomeration, but the filler-matrix layer serves as a protective barrier for the fibers under extended sliding conditions. The enhanced wear resistance of H0.75% composites is primarily due to minimized surface fragmentation and reduced asperity-induced fractures, aligning with findings from earlier studies.^42,62–64^. The HNT fillers play a vital role in enhancing wear behaviour by providing a protective layer and improving hardness.

**Fig. 24:**
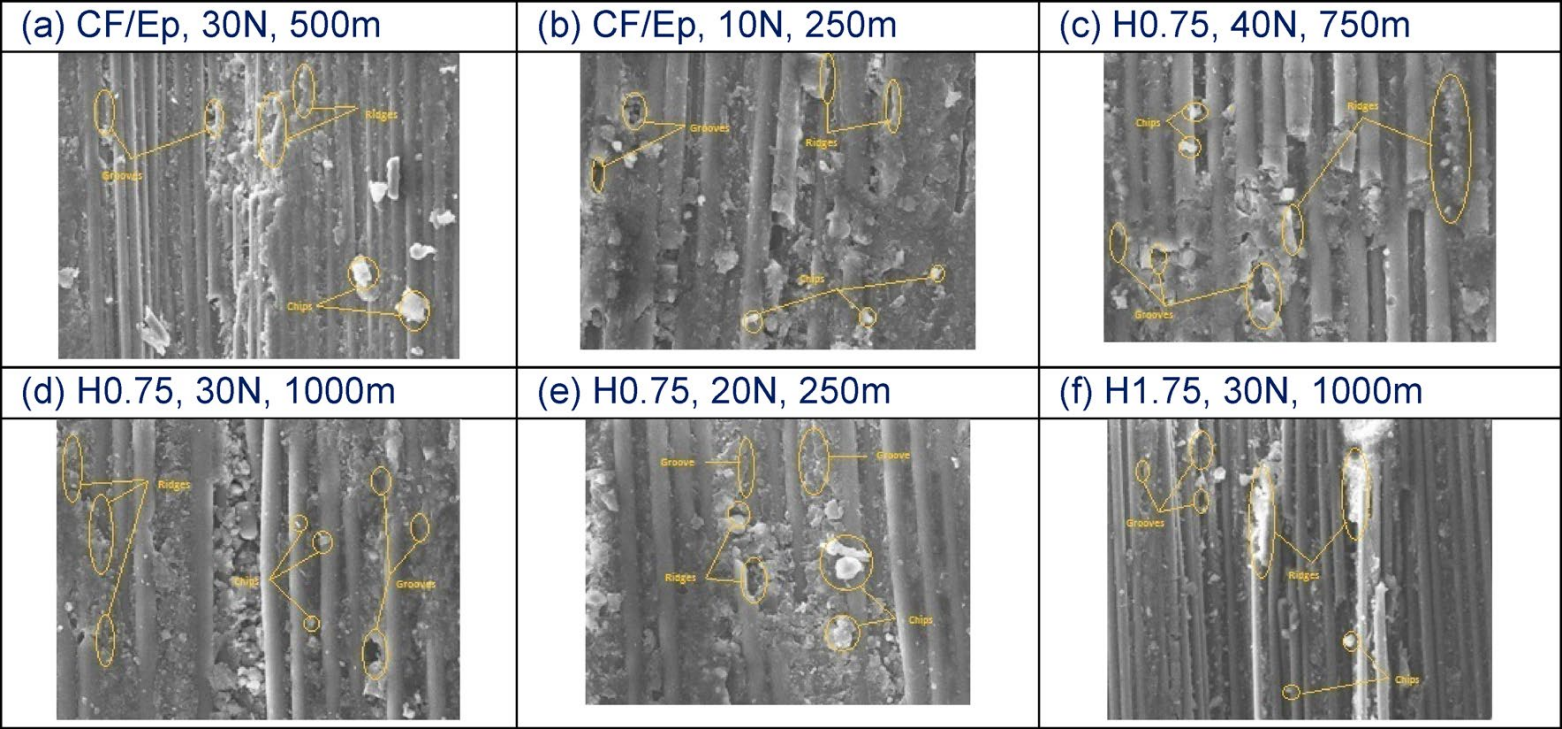
SEM worn surface of 212-HNTcomposites.

Figure 24a–f presents SEM micrographs of 212-HNT composites, illustrating uniform dispersion of HNTs and a well-bonded reinforcement–matrix interface. Wear tracks aligned with the abrading direction are clearly visible on the worn surfaces. The images suggest that the wear mechanism involves plastic deformation, leading to the formation of deep parallel grooves accompanied by wear debris. At higher loads, cracks initiate in the matrix due to stress concentrations around the sharp edges of the fillers.

## Conclusion

- In this study, carbon fabric–reinforced epoxy composites were modified with varying loadings of halloysite nanotubes (HNTs) to enhance their thermal and tribological performance. The composites were fabricated using vacuum-assisted resin transfer molding (VARTM), and a detailed evaluation of their mechanical, thermal, and wear properties was conducted. The key findings outlined below demonstrate the influence of HNT content on improving the mechanical strength, thermal stability, and wear resistance of the composites.
- The composite with 0.75 wt.% HNT exhibited the highest tensile strength, flexural strength, and interlaminar shear strength (ILSS), attributed to uniform HNT dispersion, strong filler–matrix interaction, and efficient stress transfer. However, higher HNT loadings led to agglomeration, negatively impacting mechanical performance. Notably, the H1.75% composite demonstrated the highest toughness among all formulations.
- The curing behavior, mixing uniformity, and dispersion quality of HNT fillers within the epoxy matrix and HNT–CF–Ep composites were confirmed through DSC, FTIR, and SEM analyses.
- The unfilled composites revealed a higher glass transition temperature, but the H0.75% composites demonstrated better damping characteristics. Strong temperature-strength correlations were established by the viscoelastic analysis and corroborated by changes in storage modulus. Both the H0.75% and H1.75% composites provided improved thermal stability, as demonstrated by TGA results.
- The H0.75% composites exhibited the best friction resistance and the least wear volume loss in both the dry sliding and three-body abrasion wear tests. HNT incorporation greatly improves the wear resistance of CF-Ep composites, according to ANOVA analysis, with 0.75 wt.% loading producing the best results in three-body abrasion tests.
- The findings indicate that a small addition of HNT (0.75 wt.%) significantly enhances the mechanical, thermal, and tribological properties of CF-Ep composites. Therefore, H0.75% composites are well-suited for fabricating multifunctional components in high-performance applications requiring balanced and stable performance across multiple domains.

## Data Availability

The authors confirm that the data supporting the findings of this study are available within the article.

## Abbreviations

ANOVA: Analysis of variance
c/s: Cross-section
CNT: Carbon nanotube
COF: Co-efficient of friction or µ
D: Abrading distance
DC or tanδ: Damping factor or capacity
DOE: Design of experiments
Expt.: Experimental
G-E: Glass–epoxy composites
GnP: Graphene nanoplatelets
F: Filler wt.%
IGA: Isothermal gravimetric analysis
LDPE: Low density polyethylene
LM: Loss modulus
NC: Nano clay
PMCs: Polymer matrix composites
212-HNT composites: HNT filled CF/Ep composites tested for abrasive wear using sand particle size 212 µm

## List of symbols

L: Load
RT: Room temperature
SM: Specific wear rate
S/N Ratio: Signal to noise ratio
SWR or K_s_: Specific wear rate
S/V: Surface to volume ratio
TDS: Technical datasheet
Theor.: Theoretical
T_g_: Glass transition temperature
T_m_: Melting temperature
UTL: Upper temperature limit
UTM: Ultimate testing machine
wt.%: Weight percentage
WVL or ∆V: Wear volume loss
L_30_ D_1000_ F_1.75_: Load: 30N, Abrading distance—1000 m, Filler wt.%—1.75%
L_30_ D_1000_ F_2.75_: Load: 30N, Abrading distance—1000 m, Filler wt.%—2.75
